# Comparative efficacy of virtual reality, robotics, and brain–computer interface interventions for upper limb rehabilitation after stroke: a systematic review and network meta-analysis

**DOI:** 10.3389/fneur.2026.1882841

**Published:** 2026-09-09

**Authors:** Ruichun Pang, Zhiyin Hu, Chongyang Luo, Zhongxu Liu, Shuna Zhang

**Affiliations:** 1 College of Physical Education and Health, Guangxi Normal University, Guilin, Guangxi, China; 2 School of Exercise and Health, Shanghai University of Sport, Shanghai, China

**Keywords:** stroke, upper limb rehabilitation, virtual reality, robotics, brain–computer interface, network meta-analysis, systematic review

## Abstract

**Background:**

Stroke often leads to persistent upper-limb motor impairment, which significantly impairs quality of life. Conventional physical therapy (CPT) has limitations, including insufficient intensity, limited patient engagement, and inadequate feedback. Emerging technologies such as virtual reality (VR), robotics (ROT), and brain–computer interfaces (BCI) have shown promise; however, direct comparisons among these approaches are lacking, and their relative effectiveness remains unclear.

**Objective:**

This study aimed to systematically evaluate and compare the relative effectiveness of VR, robotics, and BCI on upper limb motor function, motor performance, and activities of daily living in stroke survivors using network meta-analysis.

**Methods:**

PRISMA-NMA guidelines were followed. PubMed, Web of Science, Cochrane Library, and Embase were searched from inception to October 2025 for RCTs. Two reviewers independently screened studies, extracted data, and assessed risk of bias using RoB 2.0. A Bayesian random-effects network meta-analysis (R package gemtc) was performed to estimate relative treatment effects and calculate SUCRA values, along with sensitivity and subgroup analyses.

**Results:**

25 RCTs (1,145 stroke survivors) were included. The network evidence geometry was star-shaped, with conventional physical therapy (CPT) as the common comparator. For FMA-UE, ROT-RFE achieved the highest SUCRA ranking, although this estimate was based on a single study. ROT-CPT and ROT, supported by two and three studies respectively, provided more consistent evidence. For secondary outcomes, ROT-CPT ranked highest for MBI (SUCRA = 0.89), whereas VR-CPT ranked highest for WMFT (SUCRA = 0.59). Sensitivity analyses generally supported robustness, and subgroup analyses suggested patient characteristics may influence treatment effects.

**Conclusion:**

For improving upper-limb motor function after stroke, robotics-based interventions were supported by stronger evidence than other modalities. Specifically, ROT (SUCRA = 0.71, 3 studies) and ROT-CPT (SUCRA = 0.70, 2 studies) demonstrated consistent and clinically meaningful improvements, representing more reliable options for clinical practice. While a single robotics variant (ROT-RFE, SUCRA = 0.91) achieved a numerically higher ranking, this estimate was based on one trial and should not be interpreted as definitive evidence of superiority. VR-based interventions showed modest benefits, whereas BCI-based interventions were supported by only one eligible study with extractable data and no reliable conclusions can be drawn.

**Systematic review registration:**

https://www.crd.york.ac.uk/prospero/display_record.php?ID=CRD420251180631, identifier: CRD420251180631.

## 1 Introduction

### 1.1 Background

Stroke remains a leading global cause of long-term disability, with 55–75% of survivors experiencing upper-limb motor impairments that reduce functional independence and socioeconomic well-being (1). Conventional physical therapy (CPT), although essential, is often constrained by low intensity, poor engagement, and limited feedback (2). In recent years, virtual reality (VR), robotics (ROT), and brain–computer interfaces (BCI) have emerged as promising technologies, offering new avenues for neurorehabilitation:

VR provides immersive, task-oriented environments that enhance engagement and promote motor learning through multisensory feedback (3).

Robotics delivers high-intensity, repetitive, and precisely assisted training while allowing objective quantification of movement, facilitating activity-dependent plasticity (4).

BCI decodes motor intention to trigger external devices (e.g., functional electrical stimulation, robotics), creating a closed-loop “intention-action-feedback” circuit that reinforces cortical excitability (5).

Although each technology has been shown effective in pairwise comparisons against conventional physical therapy (CPT), direct comparisons among the three technologies or their combinations are scarce, leaving clinicians uncertain about which approach is most effective. Moreover, the influence of patient characteristics (e.g., baseline impairment, age, sex) on treatment selection remains poorly understood. Therefore, this network meta-analysis aims to integrate direct and indirect evidence to comprehensively evaluate and compare the relative effectiveness of VR, robotics, and BCI for stroke upper-limb rehabilitation.

### 1.2 Objectives

Previous meta-analyses have mainly focused on single rehabilitation technologies, such as virtual reality (VR), robotic, or brain–computer interface (BCI) interventions, without directly comparing their relative effectiveness.

To our knowledge, no previous network meta-analysis has comprehensively compared VR, robotic, and BCI interventions within a unified analytical framework for upper limb rehabilitation after stroke.

Therefore, this study aimed to compare the efficacy of different technology-assisted rehabilitation interventions for improving upper limb motor function after stroke using a Bayesian network meta-analysis.

## 2 Methods

### 2.1 Protocol registration

The study was registered in the PROSPERO database (registration number: CRD420251180631) and reported in accordance with the PRISMA-NMA guidelines.

### 2.2 Search strategy

PubMed, Web of Science, the Cochrane Library, and Embase were systematically searched from inception to October 2025. Search terms were developed using a combination of Medical Subject Headings (MeSH) and free-text terms, including “stroke,” “cerebrovascular accident,” “upper extremity,” “virtual reality,” “robotics,” “brain-computer interface,” and “randomized controlled trial.” The full search strategies for all databases (PubMed, Web of Science, Cochrane Library, and Embase) are provided in the Supplementary material. The search strategy was developed in accordance with the PRISMA-NMA guidelines and was designed to maximize sensitivity. Only studies published in English were considered eligible for inclusion.

### 2.3 Eligibility criteria (PICOS)

Participants: Adults (≥18 years) with a clinical diagnosis of ischemic or hemorrhagic stroke at any stage (acute, subacute or chronic) and documented upper-limb motor impairment (Fugl-Meyer Assessment for Upper Extremity score <66 or equivalent). Studies with mixed etiology brain injury, subarachnoid hemorrhage without parenchymal infarction, or those confined exclusively to hand or finger function were excluded. Studies published in languages other than English were excluded from the analysis.

Interventions: Upper-limb rehabilitation training in which virtual reality (VR), robotics (ROT) or brain-computer interfaces (BCI) constituted the core or primary active component. BCI was defined as a closed-loop system that decodes motor intention to trigger external devices (e.g., functional electrical stimulation, robotics, or virtual reality feedback), ensuring active patient engagement in motor training. Interventions could be delivered alone or in combination with conventional physical therapy (CPT).

Comparators: Conventional physical therapy (CPT) was defined as active rehabilitation comprising physical therapy, occupational therapy, or task-oriented upper limb training delivered at a similar total therapy dose to the intervention group. Sham interventions (e.g., sham VR, sham BCI) were also classified as CPT only when they were provided as the control condition in the included studies. Group therapy and home exercise programs were included only if they were explicitly described as the standard care control in the original trial.

Outcomes: Primary outcome: upper-limb motor function, measured by the Fugl-Meyer Assessment-Upper Extremity (FMA-UE). Secondary outcomes: motor performance, measured by the Wolf Motor Function Test (WMFT); activities of daily living, measured by the Modified Barthel Index (MBI).

Study design: Randomized controlled trials (RCTs).

#### 2.3.1 Treatment node definition and rationale

The 10 intervention nodes were defined based on the core technological component and adjunct components:

Stand-alone interventions included VR (virtual reality training without concurrent CPT) and ROT (robotics-based training without concurrent CPT). Add-on interventions included VR-CPT, ROT-CPT, and BCI-CPT. Robotics variants were defined based on specific rehabilitation paradigms: ROT-RFE (robotics with repetitive facilitative exercise, emphasizing high-frequency motor repetition), ROT-ADL (robotics with activities-of-daily-living training, focusing on functional task transfer), ROT-HEP (robotics combined with home exercise programs, addressing treatment intensity and accessibility), and ROT-ITR (robotics combined with intensive trunk rehabilitation, addressing postural control and proximal stability). This node structure reflects genuine differences in rehabilitation philosophy and delivery that may influence efficacy. However, we acknowledge that most robotics variants (RFE, ADL, HEP, ITR) were supported by single studies, limiting the reliability of separate reporting. Control interventions were categorized as CPT, which encompassed conventional physical therapy, occupational therapy, sham interventions, and home-based exercises provided as the control condition. This heterogeneity in control treatments is unavoidable given the breadth of published stroke rehabilitation trials but is a potential source of confounding in indirect comparisons (see Discussion, Transitivity section).

### 2.4 Study selection and data extraction

Two reviewers independently performed study selection. Titles and abstracts were screened first to remove obviously irrelevant records, followed by full-text assessment. A standardized data extraction form was used to collect: first author, year, country, sample size, patient characteristics (age, sex, disease duration, baseline function), intervention details (type, frequency, duration), comparator, outcome data (means and standard deviations of endpoint or change scores), and elements for risk of bias assessment. When endpoint data were not reported, they were estimated by combining baseline values and change scores where appropriate. This approach assumes a reasonable correlation between baseline and follow-up measurements and was applied consistently across studies. For studies with missing sex information for dropouts, it was assumed that the sex ratio of dropouts was the same as that of the baseline cohort. Studies that reported only medians and interquartile ranges, or only between-group effect sizes (e.g., mean difference) without providing group-specific means and standard deviations, were excluded from quantitative synthesis. A detailed breakdown of intervention components and dosage is provided in Supplementary Table S4.

### 2.5 Risk of bias assessment

Risk of bias was assessed using the Cochrane Risk of Bias 2.0 (RoB 2.0) tool, which evaluates bias across five domains: bias arising from the randomization process, bias due to deviations from intended interventions, bias due to missing outcome data, bias in measurement of the outcome, and bias in selection of the reported result. Two reviewers independently conducted the assessment, and disagreements were resolved through discussion.

### 2.6 Statistical analysis

Pairwise meta-analysis: For direct comparisons, random-effects models were used to pool mean differences (MD) and 95% confidence intervals. Heterogeneity was evaluated using the I^2^ statistic.

For studies reporting both endpoint and change scores, change scores were preferentially extracted. For studies reporting only endpoint values, the correlation between baseline and follow-up was assumed to be r = 0.5, consistent with previous NMA methodological recommendations. The standard deviation of change scores was imputed using the formula: SD_change = sqrt (SD_baseline^2^ + SD_follow-up^2^–2 × r × SD_baseline × SD_follow-up). The choice of correlation coefficient (r = 0.5) was consistent with previous NMA methodological recommendations. A sensitivity analysis using alternative correlation coefficients (r = 0.3 and r = 0.7) was performed by re-imputing change-score standard deviations from the original study data. The results remained consistent with the primary analysis (Supplementary Table S6), confirming that the conclusions were not sensitive to the choice of correlation coefficient.

Network meta-analysis: Conventional physical therapy (CPT) served as the common reference. The effect size for each intervention relative to CPT, along with its 95% credible interval (CrI), was calculated. Network evidence plots were drawn, and the surface under the cumulative ranking curve (SUCRA) was computed; higher SUCRA values indicated a greater likelihood of being the best treatment. It should be noted that SUCRA values reflect ranking probabilities rather than the magnitude or certainty of treatment effects and should therefore be interpreted in conjunction with effect sizes and their uncertainty.

The assumption of transitivity was evaluated by examining the similarity of included studies in terms of clinical and methodological characteristics, such as patient demographics, baseline severity, and intervention protocols, which were considered to support the validity of indirect comparisons. Due to the star-shaped network structure with no closed loops, statistical inconsistency could not be formally assessed. Therefore, the assumption of consistency remains untestable and should be interpreted with caution (6).

The Bayesian model was implemented using the gemtc package (R). Model specifications were as follows: likelihood = normal; link = identity; four Markov chains were run with 20,000 adaptation iterations, 50,000 burn-in iterations, and 100,000 sampling iterations with a thinning interval of 10. Non-informative prior distributions were used: a normal prior N (0, 100^2^) for treatment effects, and a uniform prior U(0, 10) for the between-study standard deviation. Convergence was assessed using the Gelman-Rubin statistic (R-hat), with values < 1.05 considered acceptable. The full analysis code and input dataset are provided in Supplementary file S1.

Sensitivity analysis: Four studies with a high risk of bias (7–10) were excluded to test the robustness of the main conclusions.

Subgroup analyses were conducted based on median values due to the lack of established clinical thresholds. These analyses were exploratory in nature and intended to generate hypotheses rather than confirm causal relationships.

Publication bias: Publication bias was not formally assessed because the number of studies per comparison was generally small and the network structure was star-shaped (<10).

## 3 Results

### 3.1 Literature screening process and results

**Figure 1:**
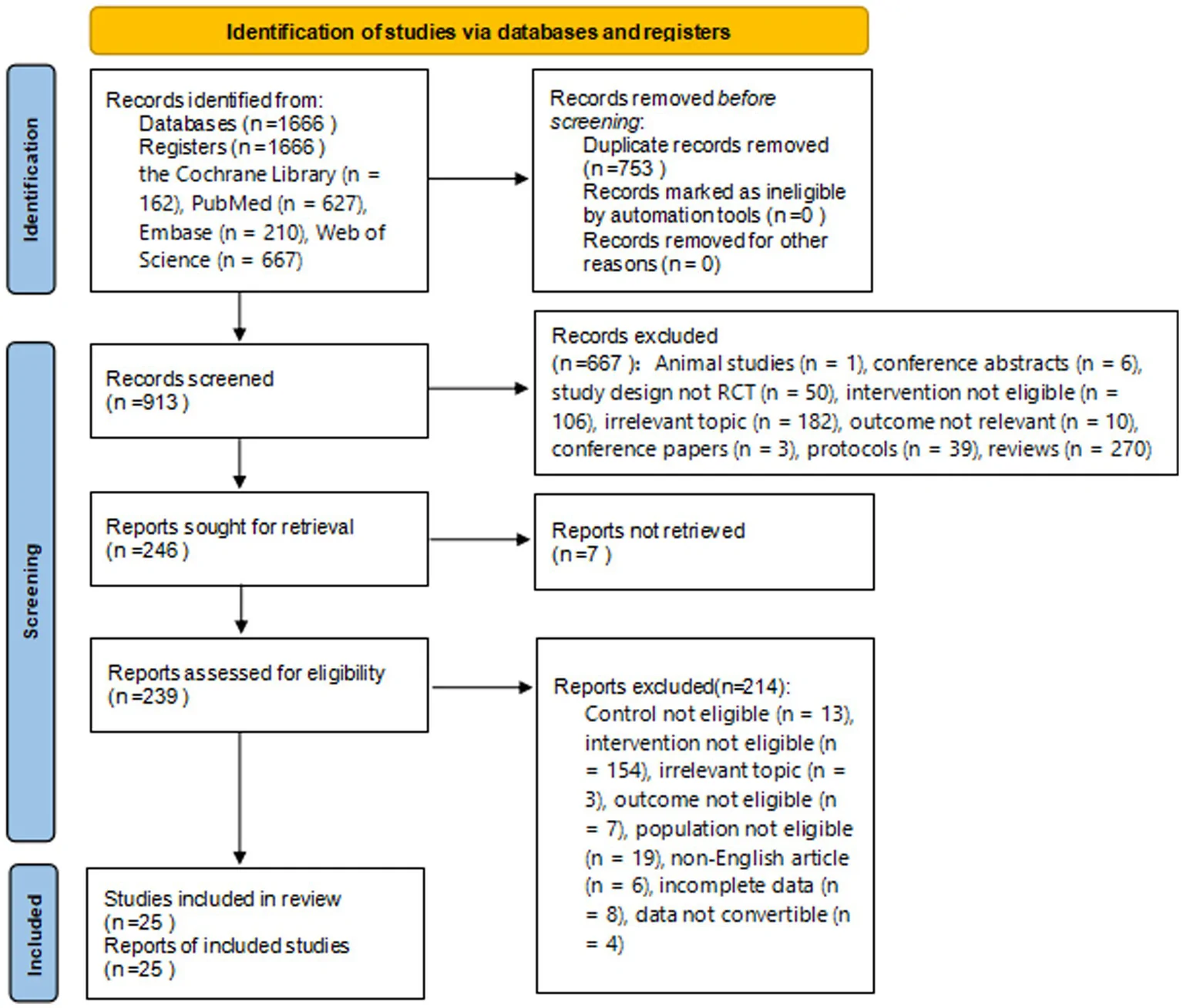
PRISMA flow diagram of the study selection process.

A total of 1,666 articles were initially identified. After removing duplicates using Endnote, 913 articles remained. Following title and abstract review, 667 articles were excluded. Seven articles were not retrieved, and 214 articles were excluded after full-text reading. Ultimately, 25 RCTs were included (PRISMA flowchart shown in Figure 1). All included studies were two-arm designs, involving a total of 1,145 stroke survivors. The literature search was completed in October 2025. Studies published after this date were not included to maintain consistency with the PROSPERO-registered protocol.

Outcome-specific network composition: Of the 25 included RCTs, 23 contributed extractable FMA-UE data (primary outcome). Wang et al. (11) was excluded from the FMA-UE network because data were reported as medians and interquartile ranges without group-specific means and standard deviations; Jo et al. (12) did not report FMA-UE and was also excluded. For secondary outcomes, 9 RCTs contributed MBI data and 3 RCTs contributed WMFT data. Detailed study-to-node allocation and outcome availability are summarized in Supplementary Table S3 and Table 1.

**Table 1:** Baseline characteristics of the 25 included randomized controlled trials.

Study	Country	Sample size (EG/CG)	Age (years, mean ± SD)	Male (%)	Intervention	Control	Intervention dose/frequency	Outcomes reported
Piron et al. (33)	Italy	18/18	66.0 ± 7.9/64.4 ± 7.9	61.1/55.6	VR	CPT	1 h/d, 5 d/wk., 4 wk	FMA-UE
Reinkensmeyer et al. (34)	USA	13/13	60 ± 10/61 ± 13	38.5/92.3	ROT	CPT	24 sessions, 3/wk., 8–9 wk	FMA-UE
Ramos-Murguialday et al. (7)	Germany	16/14	49.3 ± 12.5/50.3 ± 12.2	56.3/64.3	BCI + CPT	BCIsham+CPT	Daily training, 4 wk	FMA-UE*
Kiper et al. (9)	Italy	23/21	63.1 ± 9.5/65.5 ± 14.2	60.9/71.4	VR + CPT	CPT	1 h/d, 5 d/wk., 4 wk	FMA-UE
Shin et al. (10)	Korea	9/7	52.0 ± 11.9/46.6 ± 5.8	55.6/42.9	VR + CPT	CPT	10 sessions, 40 min/session, 2 wk	FMA-UE, MBI
Shin et al. (35)	Korea	16/16	53.3 ± 11.8/54.6 ± 13.4	68.8/81.3	VR + CPT	CPT	1 h/d, 5 d/wk., 4 wk	FMA-UE
Kim et al. (36)	Korea	12/11	56.7 ± 17.8/57.2 ± 15.0	58.3/90.9	VR + CPT	VRsham+CPT	30 min/d, 5 d/wk., 2 wk	FMA-UE, MBI
Kiper et al. (37)	Italy	68/68	62.5 ± 15.2/66.0 ± 12.9	54.4/63.2	VR + CPT	CPT	2 h/d, 5 d/wk., 4 wk	FMA-UE
Lee et al. (38)	Korea	15/15	52.1 ± 14.1/50.3 ± 11.2	53.3/73.3	ROT+CPT	CPT	1 h/d, 5 d/wk., 8 wk	FMA-UE, MBI
Oh et al. 2019 (39)	Korea	17/14	57.4 ± 12.2/52.6 ± 10.7	70.6/64.3	VR	CPT	30 min/d, 3 d/wk., 6 wk	FMA-UE
Park et al. (40)	Korea	12/13	53.5 ± 13.0/51.5 ± 16.7	58.3/61.5	VR + CPT	CPT	1 h/d, 5 d/wk., 4 wk	FMA-UE, MBI, WMFT
Carpinella et al. (41)	Italy	19/19	67.0 (58.0–70.0) / 59.0 (46.0–69.0)	52.6/52.6	ROT	CPT	45 min/d, 5 d/wk., 4 wk	FMA-UE
Budhota et al. (42)	Singapore	21/22	56.3 ± 10.4/54.6 ± 10.9	52.4/63.6	ROT+CPT	CPT	90 min/d, 3 d/wk., 6 wk	FMA-UE
Mekbib et al. (43)	China	12/11	52.2 ± 13.3/61.0 ± 7.7	75.0/72.7	VR + CPT	CPT	2 h/d, 4 d/wk., 2 wk	FMA-UE, MBI
Senocak et al. (44)	Turkey	19/22	60.2 ± 10.5/63.7 ± 9.1	52.6/63.6	ROT+ITR	CPT + ITR	2 h/d, 5 d/wk., 6 wk	FMA-UE, WMFT
Chang et al. (45)	Korea	15/15	65.2 ± 5.4/67.2 ± 5.4	46.7/46.7	ROT	CPT	30 min/d, 10 sessions, 4 wk	FMA-UE, MBI
Huang et al. (46)	China	20/20	63.3 ± 14.3/65.1 ± 6.1	65.0/55.0	VR + CPT	CPT	1 h/d, 5 d/wk., 3 wk	FMA-UE, MBI
Zhang et al. (47)	China	25/25	51.6 ± 12.3/50.3 ± 14.0	64.0/64.0	ROT+RFE	CPT	1 h/d, 5 d/wk., 4 wk	FMA-UE
Wang et al. 2024 (11)	China	150/146	60 (52–67) / 58 (52–66)	73.3/78.8	BCI + CPT	CPT	1 h/d, 5 d/wk., 4 wk	FMA-UE, WMFT
Bhattacharjee et al. (48)	India	22/22	53.3 ± 9.9/53.2 ± 10.5	59.1/54.5	ROT+ADL	CPT + ADL	1 h/d, 5 d/wk., 3 wk	FMA-UE
Ögün et al. (49)	Turkey	33/32	61.5 ± 10.9/59.8 ± 8.1	84.8/71.9	VR	VRsham+CPT	1 h/d, 3 d/wk., 6 wk	FMA-UE
Lee et al. (50)	Korea	13/13	66.5 ± 7.3/69.9 ± 7.2	76.9/61.5	VR + CPT	CPT + CPTG	1 h/d, 3 d/wk., 8 wk	FMA-UE, MBI
Jo et al. (12)	Korea	15/14	58.2 ± 10.1/60.1 ± 8.7	66.7/57.1	VR + CPT	CPT	Conventional: 30 min/d, 3 d/wk., 4 wk.; VR: 60 min/d, 5 d/wk., 4 wk	WMFT
Wolf et al. (51)	USA	47/45	59.1 ± 14.1/54.7 ± 12.2	66.0/55.6	ROT+HEP	HEP	3 h/d, 5 d/wk., 8 wk	FMA-UE, WMFT
Ballester et al. (8)	Spain	17/18	65.1 ± 10.3/61.8 ± 12.9	47.1/33.3	VR + CPT	CPT	20–60 min/d, 5 d/wk., 3 wk	FMA-UE, MBI

EG = experimental group, CG = control group, CPT = conventional physical/occupational therapy, VR = virtual reality, ROT = robotics, BCI = brain-computer interface, RFE = repetitive facilitative exercise, ITR = intensive trunk rehabilitation, ADL = activities of daily living, HEP = home exercise program, CPTG = group therapy. * Ramos-Murguialday et al. (7) used cFMA (0–54), which was converted to FMA-UE (0–66) by multiplying by 66/54. Data are presented as mean ± SD or median (interquartile range).

### 3.2 Basic characteristics of included studies

The basic characteristics of the included studies are shown in Table 1. The 25 studies were published between 2009 and 2025 and were conducted in countries including Italy, South Korea, the United States, Germany, China, and Turkey. The intervention types included: VR (2 studies), VR combined with conventional physical therapy (CPT) (9 studies), robotics (3 studies), robotics combined with conventional physical therapy (CPT) (3 studies), robotics combined with other adjunct therapies (4 studies), and BCI combined with conventional physical therapy (CPT) (2 studies). All studies used conventional physical therapy (CPT) or CPT with adjunct treatments as the control group. The average age of stroke survivors ranged from 46 to 69 years, with males comprising 40 to 78%. The baseline FMA-UE scores ranged from 7.9 to 52.3, covering mild to moderate upper limb dysfunction. A summary of clinical characteristics by intervention node is provided in Supplementary Table S3.

### 3.3 Risk of bias assessment

**Figure 2:**
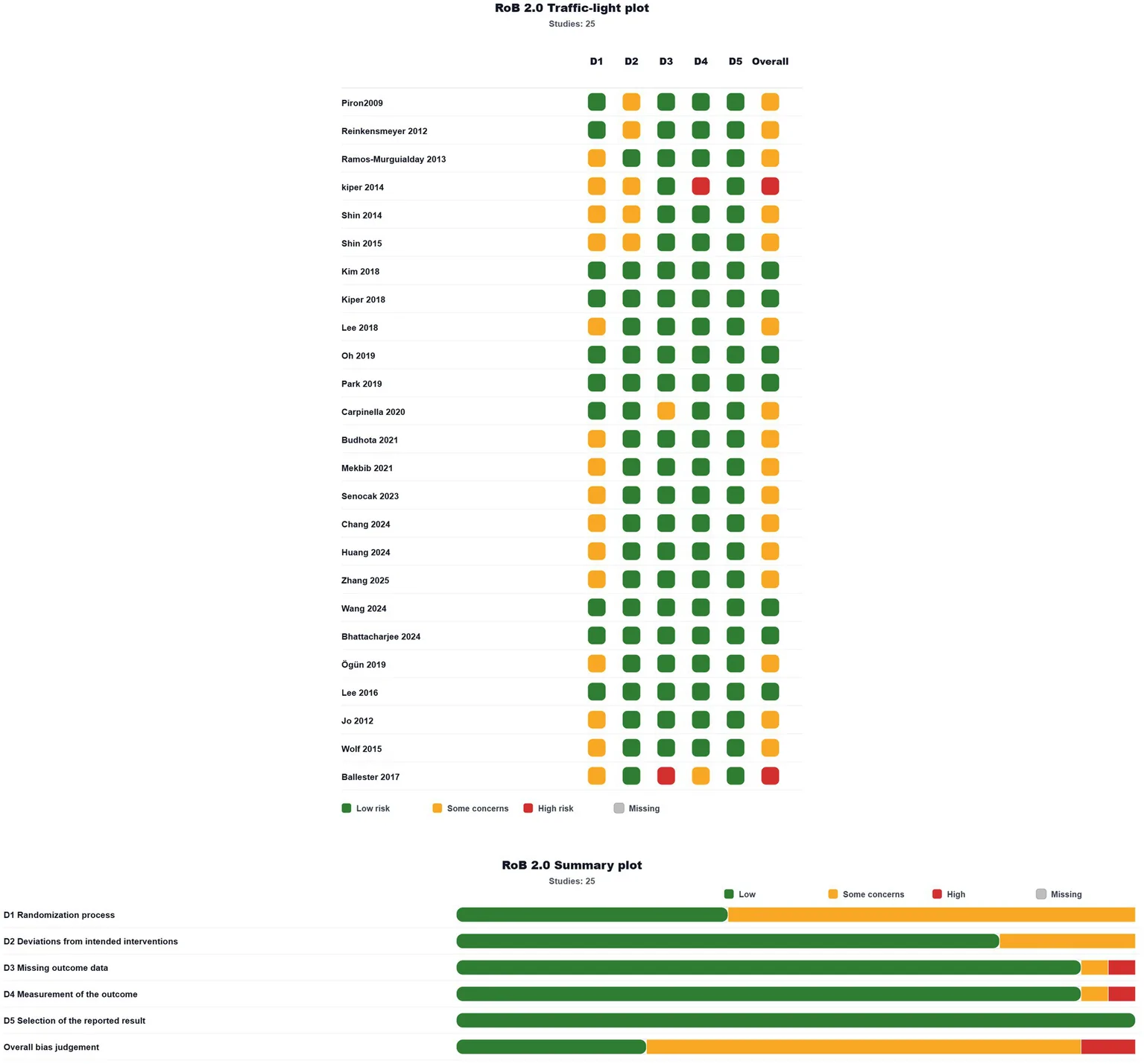
Risk of bias assessment of the included studies using the RoB 2.0 tool.

The results of the risk of bias assessment are shown in Figure 2. In the domain of random sequence generation (D1), 17 studies (68%) were at low risk, 8 studies (32%) had some concerns, and none were at high risk. In the domain of deviation from intended interventions (D2), 24 studies (96%) were at low risk, none had some concerns, and one study (10) was judged at high risk due to mismatched total therapy time between groups. In the domain of incomplete outcome data (D3), all 25 studies (100%) were at low risk. In the domain of outcome measurement bias (D4), 23 studies (92%) were at low risk, none had some concerns, and two studies (7, 8) were judged at high risk due to detection bias. In the domain of selective reporting bias (D5), 24 studies (96%) were at low risk, and one study (9) was judged at high risk due to selective reporting of outcomes. Complete domain-level RoB 2 assessments for all 25 studies are provided in Supplementary Table S5. Overall, 14 studies were rated as low risk, 7 as some concerns, and 4 as high risk.

### 3.4 Results of conventional pairwise meta-analysis

Compared with conventional physical therapy (CPT), most interventions showed improvements in FMA-UE scores. The combined effect sizes (MD) and 95% confidence intervals are shown in Table 2. Among the interventions, ROT-RFE showed the largest effect size (MD = 10.18, 95% CI: 3.25–17.28), but this result was supported by only one study. ROT (MD = 6.46, 95% CI: 0.50–12.49) and ROT-CPT (MD = 6.49, 95% CI: 0.13–12.84) demonstrated a statistically significant improvement compared with CPT, with 3 and 2 studies supporting them, respectively. VR showed an effect size of 5.20 (95% CI: 0.23–9.92), which was statistically significant but small. The remaining interventions (BCI-CPT, VR-CPT, etc.) showed no significant difference compared with CPT.

**Table 2:** Pairwise meta-analysis results for each intervention compared with CPT for the primary outcome (FMA-UE).

Intervention	MD (95% CI)	Statistical significance	Number of studies
ROT-RFE	10.18 (3.25, 17.28)	Significant	1
ROT	6.46 (0.50, 12.49)	Significant	3
ROT-CPT	6.49 (0.13, 12.84)	Significant	2
VR	5.20 (0.23, 9.92)	Significant	3
ROT-ADL	3.54 (−3.90, 10.96)	Non-significant	1
VR-CPT	2.05 (−1.56, 5.08)	Non-significant	10
BCI-CPT	1.25 (−9.09, 11.28)	Non-significant	1
ROT-HEP	0.93 (−17.69, 19.23)	Non-significant	1
ROT-ITR	0.06 (−10.90, 10.98)	Non-significant	1
CPT	Reference	—	23

Only 23 studies that reported FMA-UE were included. Wang et al. (11) was excluded from the FMA-UE analysis because its data were reported as medians (95% CI) and could not be converted to mean ± standard deviation. Jo et al. (12) did not report FMA-UE and was therefore also excluded.

MD: Mean difference. A positive value indicates that the intervention is superior to conventional physical therapy (CPT).

95% CI: 95% confidence interval. An interval that does not include 0 indicates a statistically significant difference.

Several interventions (ROT-RFE, BCI-CPT, ROT-ADL, ROT-HEP, ROT-ITR) are supported by only one study, providing limited evidence.

ROT-CPT and ROT are supported by two and three studies, respectively, and are therefore relatively more robust.

The heterogeneity parameter sd.d was 2.45 (0.18–6.54), indicating mild to moderate heterogeneity between studies.

Heterogeneity assessment: For direct comparisons with at least two studies, heterogeneity was evaluated using the I^2^ statistic.

ROT vs. CPT (3 studies): I^2^ = 0% (*p* = 0.63), indicating no heterogeneity.
VR vs. CPT (3 studies): I^2^ = 0% (*p* = 0.69), no heterogeneity.
For VR-CPT vs. CPT (10 studies), the heterogeneity test yielded Q = 18.86, df = 9, *p* = 0.026, and I^2^ = 52.3%. The I^2^ indicates substantial heterogeneity, which should be taken into account when interpreting the results.

The remaining comparisons (ROT-RFE, ROT-CPT, ROT-ADL, ROT-HEP, ROT-ITR, BCI-CPT) each contained only one study, precluding heterogeneity estimation.

The minimal clinically important difference (MCID) for the FMA-UE in stroke survivors has been reported to range from 5.2 to 7.2 points (13, 14). The point estimates for ROT-RFE (10.18 points), ROT (6.46 points), and ROT-CPT (6.49 points) exceeded the MCID threshold. However, the 95% confidence intervals for ROT and ROT-CPT extended below the MCID threshold (lower bounds: 0.50 and 0.13, respectively), indicating that the clinical significance of these estimates remains uncertain (15, 16). The overall network-level heterogeneity parameter, sd.d, was 2.45 (0.18–6.54), indicating mild to moderate heterogeneity across the entire network.

### 3.5 Results of network meta-analysis

#### 3.5.1 Evidence network plot

**Figure 3:**
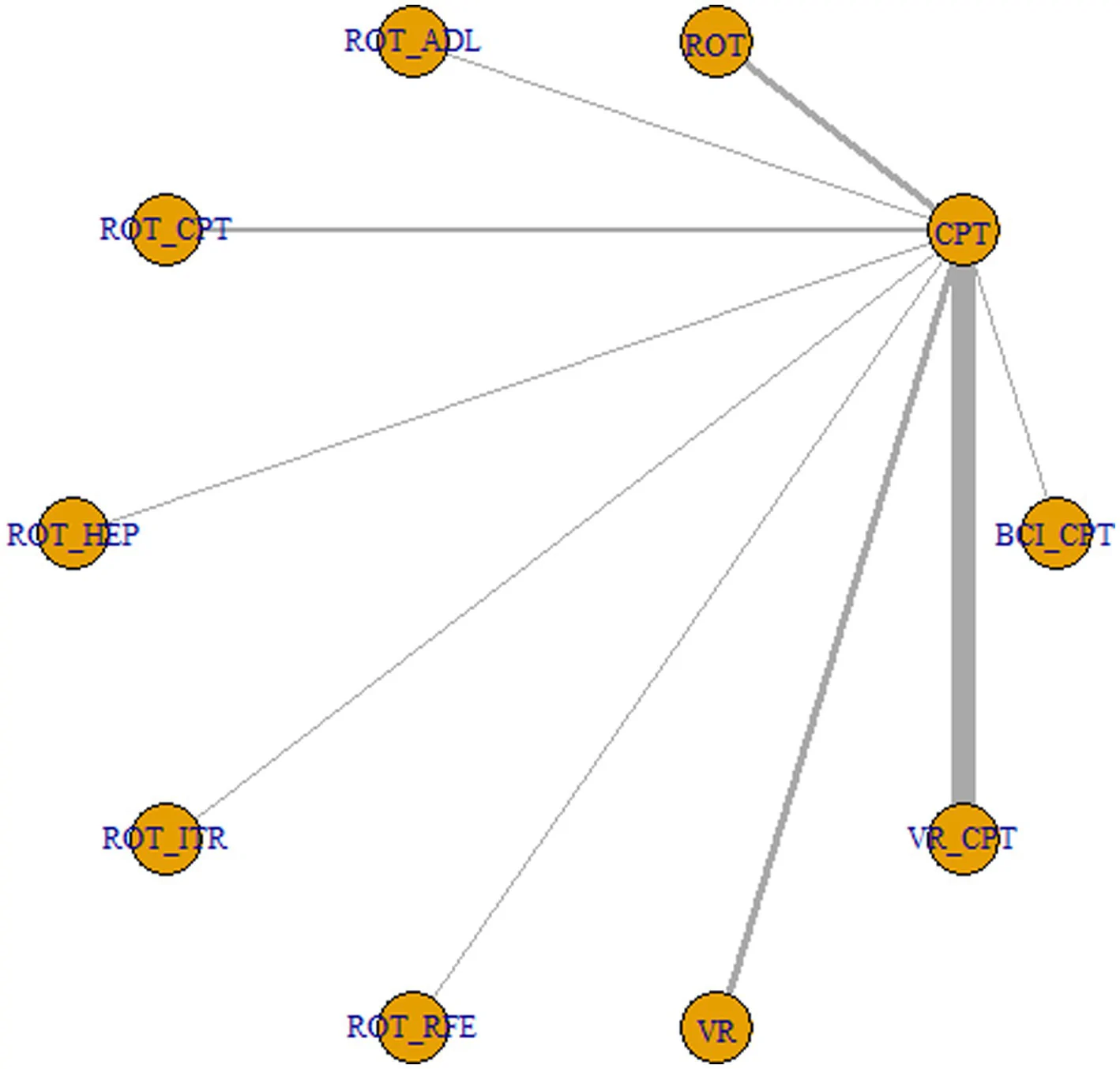
Network evidence plot of the treatment comparisons for the primary outcome (FMA-UE).

The network evidence plot (Figure 3) shows that all interventions were compared with conventional physical therapy (CPT), forming a star-shaped network. A total of 25 studies were included, involving 10 different interventions: BCI-CPT, ROT, ROT-ADL, ROT-CPT, ROT-HEP, ROT-ITR, ROT-RFE, VR, VR-CPT, and CPT. Because the network was star-shaped and all interventions were compared only with CPT, the estimates from the network meta-analysis were highly similar to those from the direct pairwise meta-analysis.

#### 3.5.2 Primary outcome (FMA-UE)

**Figure 4:**
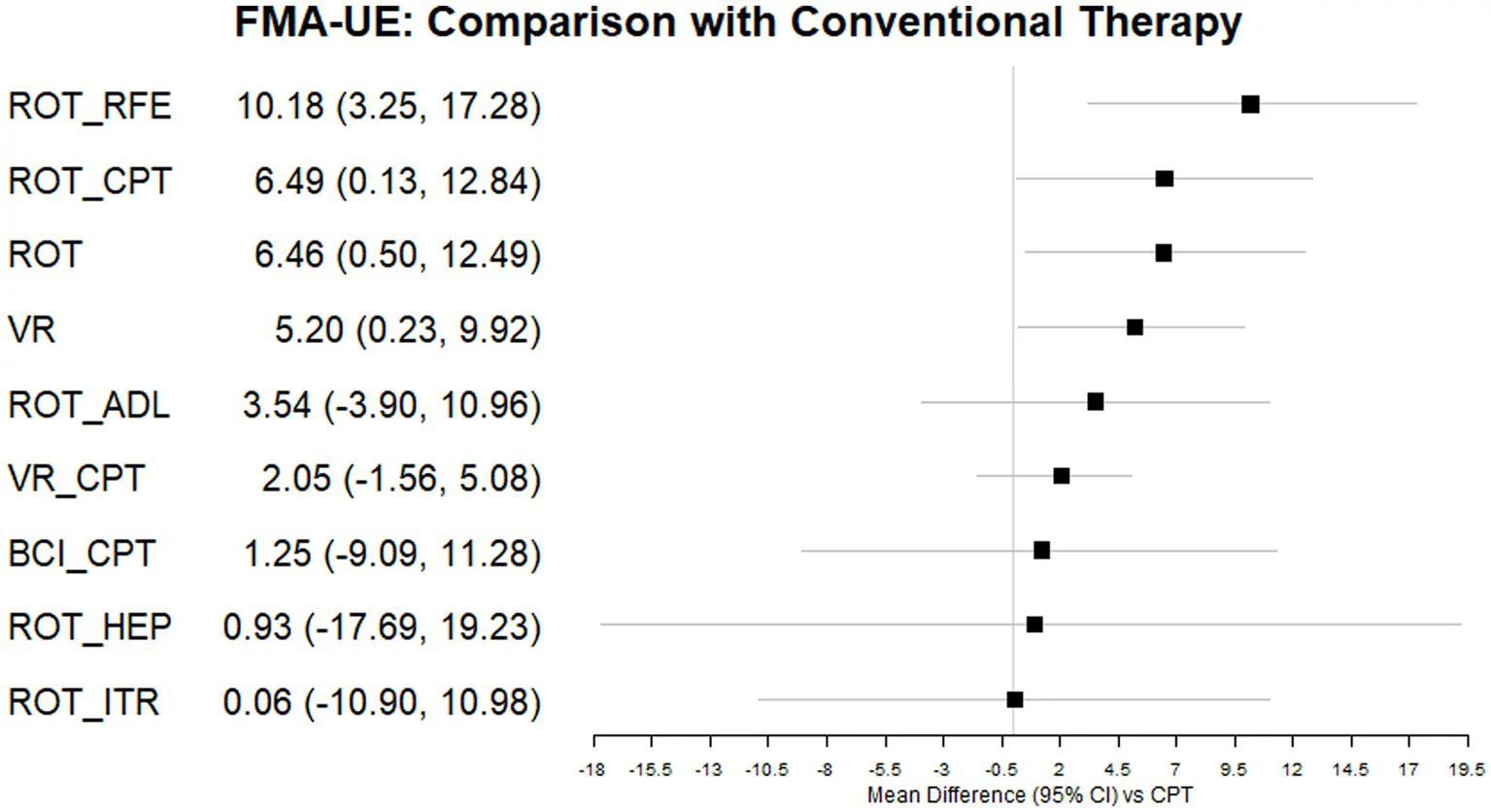
Forest plot of network meta-analysis results for FMA-UE (primary outcome).

**Figure 5:**
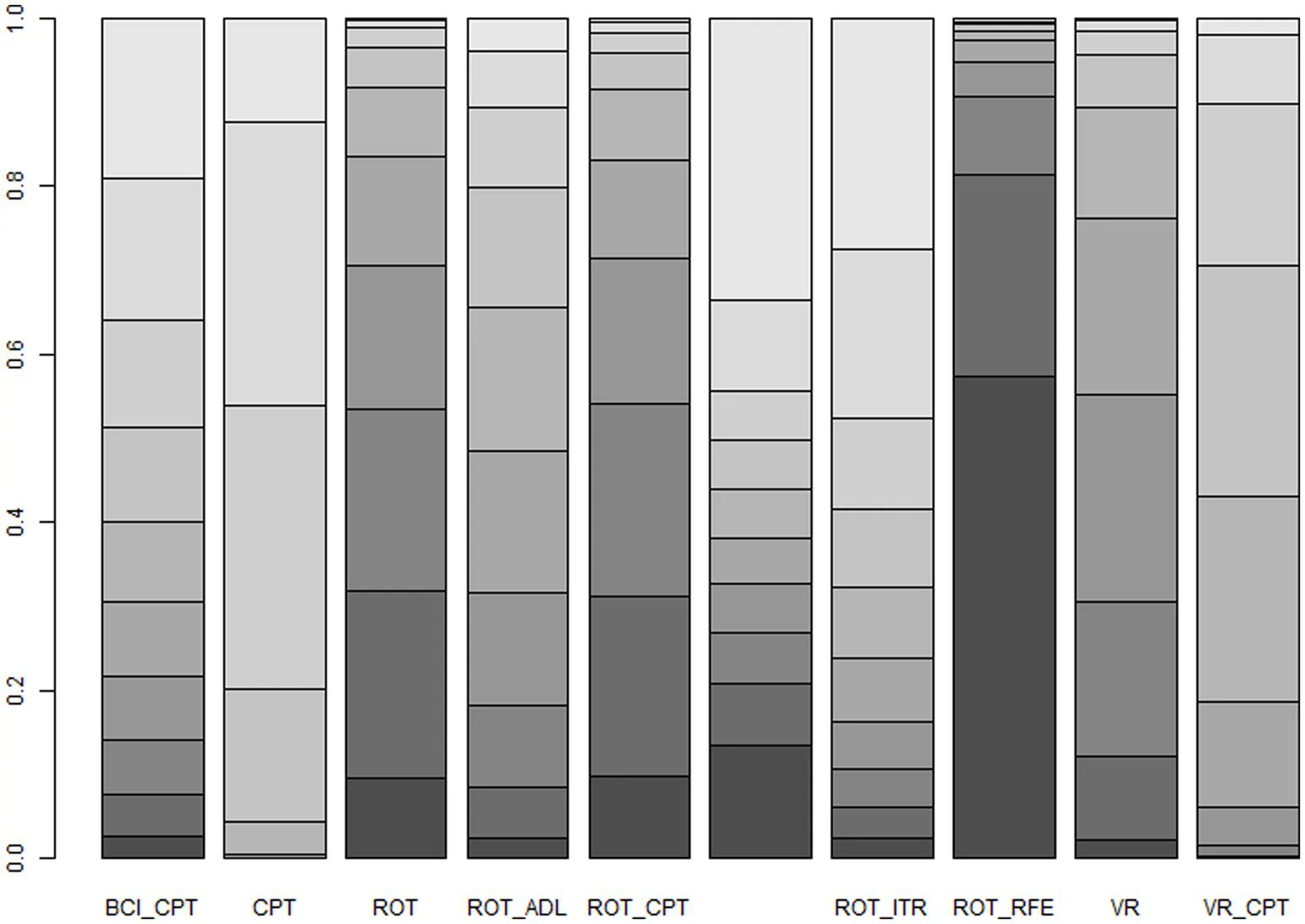
SUCRA ranking bar chart for the primary outcome (FMA-UE).

The results of the network meta-analysis (Table 3; Figures 4, 5) show that, in improving upper limb motor function, although ROT-RFE had the highest SUCRA value (0.91), this estimate was derived from a single study and should therefore be interpreted cautiously. In contrast, ROT (SUCRA = 0.71, MD = 6.46, 95% CrI: 0.50–12.49) and ROT-CPT (SUCRA = 0.70, MD = 6.49, 95% CrI: 0.13–12.84) were supported by three and two studies, respectively, indicating more consistent evidence. VR had a SUCRA of 0.62, with an effect size of 5.20 (95% CrI: 0.23–9.92), supported by 3 studies. The remaining interventions (BCI-CPT, ROT-ADL, ROT-HEP, ROT-ITR) all had SUCRA values below 0.50 and showed no significant difference compared with CPT, with most being supported by only 1–2 studies. VR-CPT also had a SUCRA below 0.50 (0.36) and did not show a significant difference (MD = 2.05, 95%CrI:−1.56–5.08), despite being supported by 10 studies. The model showed good convergence (all R-hat ≈ 1). The ranking probability plot (Figure 5) visually displays the probability of each intervention being ranked in different positions, further confirming the SUCRA ranking results, where ROT-RFE had the highest probability (about 90%) of being the most effective intervention, and CPT had the lowest probability of being the best treatment. A complete league table of all pairwise comparisons (mean differences and 95% credible intervals) is provided in Supplementary Table S1.

**Table 3:** Network meta-analysis results for the primary outcome (FMA-UE).

Intervention	SUCRA value	Rank	MD (95% CrI)	Statistical significance
ROT-RFE	0.91	1	10.18 (3.25, 17.28)	Significant
ROT	0.71	2	6.46 (0.50, 12.49)	Significant
ROT-CPT	0.70	3	6.49 (0.13, 12.84)	Significant
VR	0.62	4	5.20 (0.23, 9.92)	Significant
ROT-ADL	0.49	5	3.54 (−3.90, 10.96)	Non-significant
ROT-HEP	0.39	6	0.93 (−17.69, 19.23)	Non-significant
VR-CPT	0.36	7	2.05 (−1.56, 5.08)	Non-significant
BCI-CPT	0.35	8	1.25 (−9.09, 11.28)	Non-significant
ROT-ITR	0.29	9	0.06 (−10.90, 10.98)	Non-significant
CPT	0.19	10	Reference	—

MD = mean difference. A positive value indicates that the intervention is superior to conventional physical therapy (CPT). 95% CrI = 95% confidence interval. If the interval does not include 0, the difference is statistically significant. ROT-RFE, BCI-CPT, ROT-ADL, ROT-HEP, and ROT-ITR are each supported by only one study, providing limited evidence. Higher SUCRA values indicate a greater probability of being the best treatment.

#### 3.5.3 Secondary outcomes (MBI, WMFT)

**Figure 6:**
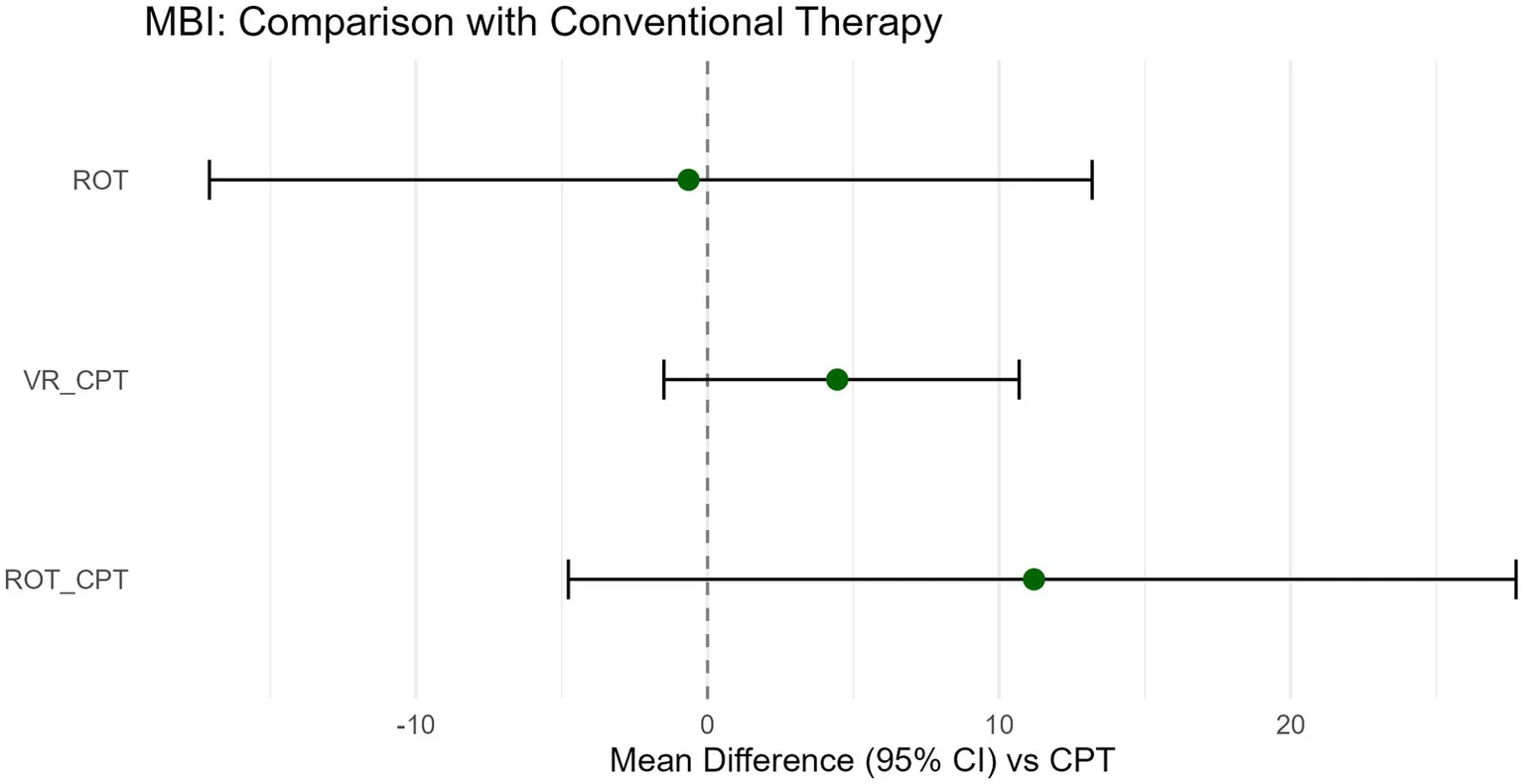
Forest plot of network meta-analysis results for MBI (secondary outcome).

MBI: Nine studies provided data, involving ROT-CPT, VR-CPT, ROT, and CPT. The SUCRA ranking (Table 4; Figure 6) showed that ROT-CPT ranked highest based on SUCRA (0.89), but the effect size did not reach statistical significance (MD = 11.20, 95% CrI: −4.77 to 27.74); VR-CPT ranked second (SUCRA = 0.64, MD = 4.45, 95% CrI: −1.50 to 10.69); ROT and CPT had similar SUCRA values (0.25 vs. 0.23). ROT-CPT was supported by only 2 studies, while VR-CPT was supported by 5 studies.

**Table 4:** Network meta-analysis results for the Modified Barthel Index (MBI).

Intervention	SUCRA value	Rank	MD (95% CrI)	Statistical significance
ROT-CPT	0.89	1	11.20 (−4.77, 27.74)	Non-significant
VR-CPT	0.64	2	4.45 (−1.50, 10.69)	Non-significant
ROT	0.25	3	−0.65 (−17.09, 13.19)	Non-significant
CPT	0.23	4	Reference	—

**Figure 7:**
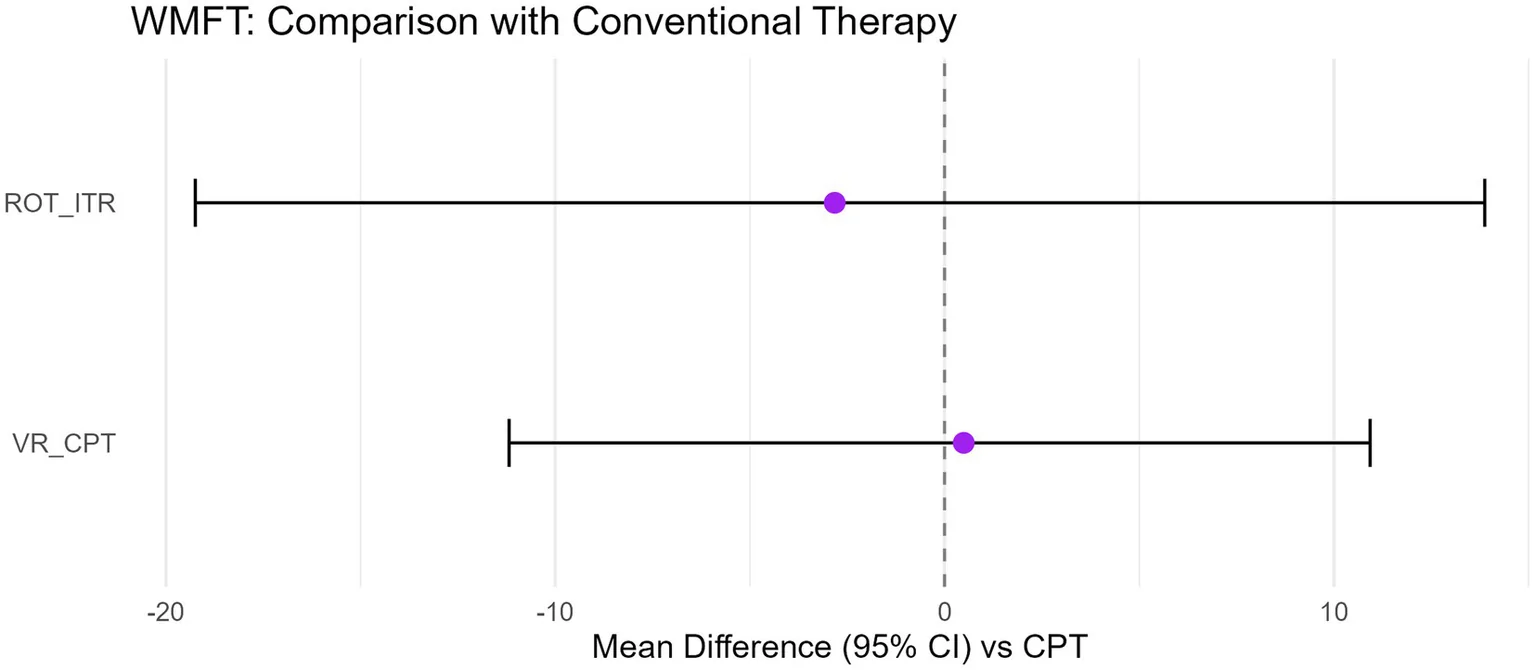
Forest plot of network meta-analysis results for WMFT (secondary outcome).

WMFT: Only 3 studies provided data, involving VR-CPT, ROT-ITR, and CPT. The SUCRA ranking (Table 5; Figure 7) showed that VR-CPT was slightly better than CPT (0.59 vs. 0.55), but the effect size was not statistically significant (MD = 0.49, 95% CrI: −11.18 to 11.00); ROT-ITR had a SUCRA of 0.36, with an effect size of −2.82 (95% CrI: −19.24 to 13.87). Due to the very small number of studies, the results for this outcome should be considered exploratory.

**Table 5:** Network meta-analysis results for the Wolf Motor Function Test (WMFT).

Intervention	SUCRA value	Rank	MD (95% CrI)	Statistical significance
VR-CPT	0.59	1	0.49 (−11.18, 11.00)	Non-significant
CPT	0.55	2	Reference	—
ROT-ITR	0.36	3	−2.82 (−19.24, 13.87)	Non-significant

A total of 9 studies were included for MBI, and 3 studies for WMFT. MD = mean difference. A positive value indicates that the intervention is superior to conventional physical therapy (CPT). MD (95% CrI) = 95% confidence interval. ROT-CPT had the highest SUCRA for MBI, but the effect did not reach statistical significance; ROT-CPT was supported by only 2 studies. For WMFT, none of the interventions showed statistically significant differences, and the number of studies was very small (VR-CPT: 2 studies; ROT-ITR: only 1 study); therefore, the results should be considered exploratory.

Higher SUCRA values indicate a greater probability of being the best treatment, but the evidence strength is limited.

Sensitivity analyses using alternative correlation coefficients (r = 0.3 and r = 0.7) were performed by re-imputing change-score standard deviations from the original study data. The SUCRA rankings remained consistent across all three values (Supplementary Table S6), with ROT-RFE, ROT, and ROT-CPT occupying the top three positions in all cases, confirming that treatment rankings were not materially affected by the choice of correlation coefficient.

### 3.6 Sensitivity analysis

To test the robustness of the main conclusions, four studies with high risk of bias were excluded. The reasons for exclusion were as follows:

Ramos-Murguialday et al. (7): used the cFMA (0–54) scale, which required conversion to FMA-UE (0–66); the conversion may have introduced measurement bias.

Ballester et al. (8): outcome assessors were not blinded (high risk of detection bias).

Kiper et al. (9): selective reporting bias (only partial outcome data available).

Shin et al. (10): mismatched total therapy time between groups (experimental group received 40 min/session, control 20 min/session), leading to performance bias.

After excluding these four high-risk studies, the SUCRA rankings and effect size estimates remained directionally consistent with the primary analysis (Table 6). ROT-RFE remained highest-ranked (SUCRA = 0.90 vs. 0.91), ROT and ROT-CPT maintained comparable rankings (0.691–0.688 vs. 0.70–0.71). This directional consistency suggests that the primary findings are not substantially confounded by the excluded studies’ risk of bias.

**Table 6:** Sensitivity analysis results after excluding four studies with a high risk of bias (7–10).

Intervention	SUCRA value	Rank	MD (95% CrI)	Statistical significance	Main analysis SUCRA
ROT-RFE	0.901	1	10.25 (3.01, 17.73)	Significant	0.909
ROT	0.691	2	6.42 (0.25, 12.74)	Significant	0.706
ROT-CPT	0.688	3	6.42 (−0.26, 12.96)	Non-significant	0.705
VR	0.601	4	5.12 (−0.48, 10.10)	Non-significant	0.622
ROT-ADL	0.480	5	3.64 (−4.24, 11.47)	Non-significant	0.489
ROT-HEP	0.368	6	0.80 (−18.49, 19.33)	Non-significant	0.386
VR-CPT	0.322	7	1.69 (−2.71, 5.18)	Non-significant	0.364
ROT-ITR	0.274	8	−0.10 (−11.53, 10.93)	Non-significant	0.287
CPT	0.175	9	Reference	—	0.185

The sensitivity analysis excluded four studies with a high risk of bias: Ramos-Murguialday (7), Ballester (8), Kiper (9), and Shin (10). Higher SUCRA values indicate a greater probability of being the best treatment. MD = mean difference. A positive value indicates that the intervention is superior to conventional physical therapy (CPT). 95% CrI = 95% credible interval. The main analysis SUCRA values are taken from Table 3 for comparison. Compared with the main analysis, the SUCRA rankings of all interventions remained unchanged; ROT-RFE, ROT, and ROT-CPT still occupied the top three positions, indicating directional consistency with the primary findings. BCI-CPT appeared only in the excluded studies and was therefore not included in the sensitivity analysis.

However, the evidence base for core interventions remains limited: most nodes are supported by 1–3 studies. Therefore, the term ‘robust’ should be interpreted cautiously. The sensitivity analysis provides reassurance regarding directional consistency but does not materially increase confidence in the point estimates given the small number of studies per node. This limitation is consistent with concerns raised in the literature that sensitivity analyses may overstate robustness when the evidence base remains sparse (17).

Dose-matched sensitivity analysis: To assess the potential confounding effect of unequal total therapy time between intervention and control groups, we performed an additional sensitivity analysis restricted to studies with comparable total therapy time (defined as ≤15% difference). This analysis excluded Shin et al. (10) due to imbalanced therapy time and included 22 studies from the FMA-UE network. In this dose-matched subset, the top three interventions (ROT-RFE, ROT-CPT, and ROT) remained unchanged, with ROT-RFE ranked first (SUCRA = 0.907, MD = 10.20, 95% CrI: 3.51 to 16.82), followed by ROT-CPT (SUCRA = 0.699, MD = 6.53, 95% CrI: 0.25 to 12.73) and ROT (SUCRA = 0.694, MD = 6.43, 95% CrI: 0.43 to 12.31) (see Supplementary Table S7 for detailed results).

### 3.7 Subgroup analysis

Subgroup analyses suggested that patient characteristics may influence treatment effects, although overall patterns remained broadly consistent (Table 7).

**Table 7:** Subgroup analysis SUCRA values for the primary outcome (FMA-UE) across different patient characteristics.

Intervention	Main analysis	Low baseline (FMA < 36)	High baseline (FMA ≥ 36)	Low male proportion (<60.9%)	High male proportion (≥60.9%)	Young age (<59 years)	Old age (≥59 years)	Low dose (<0.875 h)	High dose (≥0.875 h)	Short duration (<4 weeks)	Long duration (≥4 weeks)
ROT-RFE	0.91	0.84	—	—	0.84	0.83	—	—	0.85	—	0.89
ROT	0.71	0.70	—	0.77	0.46	—	0.83	0.77	—	—	0.77
ROT-CPT	0.70	—	0.86	0.60	0.70	0.69	—	0.74	0.58	—	0.69
VR	0.62	—	0.75	0.58	0.60	0.48	0.76	0.45	0.64	0.60	0.64
ROT-ADL	0.49	0.53	—	0.54	—	0.52	—	0.50	—	0.54	—
ROT-HEP	0.39	0.40	—	—	0.37	0.41	—	—	0.40	—	0.38
VR-CPT	0.36	0.51	0.28	0.48	0.31	0.40	0.41	0.31	0.46	0.74	0.16
BCI-CPT	0.35	0.41	—	0.40	—	0.40	—	—	—	—	0.37
ROT-ITR	0.29	0.35	—	0.35	—	—	0.30	—	0.32	—	0.31
CPT	0.19	0.28	0.11	0.27	0.20	0.29	0.19	0.22	0.25	0.12	0.29

Subgroup analyses are exploratory. All SUCRA values for ROT-RFE are based on a single study (47) and should be interpreted with caution.

Across stratifications by baseline motor function, age, and sex distribution, robot-assisted interventions (ROT and ROT-CPT) generally ranked higher, indicating relatively stable efficacy across different patient subgroups. In contrast, VR-based interventions tended to show comparatively better rankings under conditions of shorter duration or lower training dose, although this pattern should be interpreted cautiously given the exploratory nature of these analyses.

In subgroup analyses stratified by training dose and intervention duration, ROT and ROT-CPT showed higher SUCRA rankings in higher-dose arms compared to lower-dose arms. However, these subgroup patterns should be interpreted with caution for several reasons: (1) subgroups were defined post-hoc using median splits rather than predefined clinical thresholds; (2) several nodes contained insufficient studies for stable subgroup estimates; (3) treatment intensity may be confounded with other unmeasured factors such as study quality or patient selection. These findings are exploratory and should not be interpreted as evidence of a causal dose–response relationship pending confirmation in within-study subgroup analyses from primary trials. It is important to note that ROT-RFE was supported by only a single study and was therefore not included in subgroup comparisons. Any apparent subgroup patterns involving this intervention should be interpreted with caution and are not considered robust.

Overall, these subgroup findings should be regarded as exploratory, given the limited number of studies within each subgroup and the potential influence of residual confounding factors.

### 3.8 Publication bias

To assess publication bias, we attempted to generate a comparison-adjusted funnel plot using the funnel function in the gemtc package. However, because the network was star-shaped (all comparisons involved CPT) and the number of studies per direct comparison was small (most comparisons had only 1–3 studies), funnel plot asymmetry tests have very low power and may produce misleading results (18). Therefore, we refrained from presenting funnel plots. Instead, sensitivity analyses excluding high-risk studies were performed, and the results remained unchanged, suggesting that the primary findings were directionally consistent after exclusion of high-risk studies, although potential small-study effects cannot be ruled out.

### 3.9 GRADE evidence quality

The GRADE evidence quality for the primary outcome (FMA-UE) was assessed for the comparisons with at least two studies. For ROT vs. CPT (3 studies, I^2^ = 0%), the evidence was rated as moderate (downgraded one level for risk of bias due to lack of blinding). For VR vs. CPT (3 studies, I^2^ = 0%), evidence was rated as low (downgraded for risk of bias and imprecision because the 95% CI included values close to zero). For VR-CPT vs. CPT (10 studies, I^2^ = 52.3%), evidence was rated as low (downgraded for heterogeneity and potential publication bias due to small-study effects). The single-study comparisons (ROT-RFE, ROT-ADL, ROT-HEP, ROT-ITR, and BCI-CPT) were rated very low due to very serious imprecision (only one study) and risk of bias. ROT-CPT, supported by two studies, was rated low due to serious imprecision and risk of bias. A detailed GRADE evidence profile is provided in Supplementary Table S2.

## 4 Discussion

Overall, robotic interventions demonstrated stronger and more consistent evidence than other technologies for improving upper-limb motor function after stroke. However, the magnitude of benefit and the reliability of these rankings require cautious interpretation due to several important limitations in the current evidence base.

Among the included interventions, robotic repetitive facilitative exercise (ROT-RFE) achieved the highest SUCRA ranking. However, this result was derived from a single study, rendering the estimate unstable and susceptible to overestimation. Evidence from single-study nodes lacks robustness, and therefore, the apparent superiority of ROT-RFE should be interpreted as hypothesis-generating rather than conclusive. In contrast, robotic interventions supported by multiple studies, such as ROT and ROT combined with conventional therapy (ROT-CPT), demonstrated more consistent and clinically meaningful effects, suggesting a more reliable evidence base. These findings are consistent with previous systematic reviews demonstrating that robot-assisted therapy improves upper limb function after stroke (19).

The potential advantages of robotic interventions may be explained by their ability to deliver high-intensity, repetitive, and task-specific training, which are critical drivers of activity-dependent neuroplasticity. Repetitive motor practice has been shown to facilitate Hebbian learning mechanisms and promote cortical reorganization, thereby enhancing motor recovery after stroke (20, 21).

VR-based interventions demonstrated modest improvements in upper limb motor function. This finding is consistent with Cochrane reviews and recent meta-analyses reporting small-to-moderate effect sizes for VR in stroke rehabilitation (22, 23). The therapeutic benefits of VR are likely mediated by multisensory feedback, enhanced patient engagement, and task-oriented training, all of which contribute to motor learning and neuroplasticity (24).

In contrast, the evidence supporting BCI-based interventions is severely limited. Only one eligible study with extractable FMA-UE data (7) contributed to the primary network analysis. A larger BCI trial [(11), *n* = 300] could not be included because outcome data were reported as medians with interquartile ranges without group-specific means and standard deviations, precluding quantitative synthesis. Although the Ramos-Murguialday trial reported some improvements, the associated credible intervals remained wide, and the overall evidence base is insufficient to establish clinical recommendations. Systematic reviews have also highlighted the heterogeneity and limited robustness of current BCI evidence in stroke rehabilitation (25). Therefore, BCI interventions should be considered investigational, pending high-quality trials with standardized outcome reporting that can be incorporated into evidence synthesis.

Importantly, the network structure in this study was star-shaped, with all interventions compared only against conventional physical therapy (CPT). As a result, no closed loops were available to assess statistical inconsistency. Although the assumption of transitivity was evaluated based on the similarity of clinical and methodological characteristics across studies, the absence of direct head-to-head comparisons limits the strength of indirect inferences. While we evaluated clinical similarity across nodes, several features suggest that transitivity assumptions may be partially violated in specific comparisons:

Baseline severity heterogeneity: Baseline FMA-UE ranged from 7.9 to 52.3 across studies (Supplementary Table S3). This represents a span of 44.4 points—encompassing mild, moderate, and severe upper-limb impairment. Treatment effects may systematically differ across this spectrum (i.e., a meaningful interaction by baseline severity). For example, ROT studies had mean baseline FMA = 31.2 while VR-CPT included studies with baseline FMA as low as 7.9 and as high as 52.3. Indirect comparisons between ROT and VR therefore incorporate this baseline imbalance.

Stroke chronicity reporting: Stroke stage (acute/subacute/chronic) was not systematically reported across all studies, precluding quantitative assessment of whether nodes differed in patient chronicity. If some nodes preferentially enrolled acute patients and others chronic patients, this unobserved confounding could introduce bias in indirect estimates.

Control treatment heterogeneity: The ‘CPT’ comparator ranged from structured physical therapy (defined frequency and duration) to home exercise programs to sham interventions. This heterogeneity in reference treatment intensity could systematically bias indirect comparisons if intervention nodes preferentially compared against stronger or weaker controls.

The validity of indirect comparisons relies on the similarity of effect modifier distributions across comparisons (26). These transitivity concerns do not invalidate the network structure but highlight that some indirect comparisons—particularly between nodes with divergent baseline characteristics (e.g., ROT vs. VR-CPT)—should be interpreted with caution. Direct head-to-head trials would be needed to confirm indirect estimates in these high-discordance comparisons. Therefore, the findings should be interpreted with caution.

To further contextualize these findings, the interventions in this NMA can be stratified into three evidence tiers based on study support and consistency:

Tier 1 – Robust Evidence (≥3 studies with consistent effect directions): ROT (3 studies, SUCRA = 0.71, MD = 6.46, 95% CrI: 0.50–12.49) and VR (3 studies, SUCRA = 0.62, MD = 5.20, 95% CrI: 0.23–9.92) demonstrated consistent improvements in FMA-UE with low heterogeneity (I^2^ = 0%). These interventions provide the most reliable evidence base for clinical decision-making.

Tier 2 – Moderate Evidence (2 studies): ROT-CPT (2 studies, SUCRA = 0.70, MD = 6.49, 95% CrI: 0.13–12.84) and VR-CPT (10 studies, SUCRA = 0.36, MD = 2.05, 95% CrI: −1.56 to 5.08) showed effect estimates comparable to Tier 1 but with more limited evidence base (for ROT-CPT) or inconsistent effect direction (for VR-CPT).

Tier 3 – Limited Evidence (1 study each): Five intervention nodes—ROT-RFE, ROT-ADL, ROT-HEP, ROT-ITR, and BCI-CPT—were each supported by a single RCT. While ROT-RFE achieved the highest SUCRA value (0.91), this ranking reflects the point estimate from one trial and should not be interpreted as definitive evidence of superiority. These single-study estimates are vulnerable to sampling variation and publication bias; their apparent ranking positions are exploratory and hypothesis-generating rather than confirmatory.

Clinical implications should prioritize evidence quality over SUCRA rankings: Tier 1 interventions provide the strongest basis for recommendation, while Tier 2 findings warrant cautious interpretation, and Tier 3 estimates should not guide clinical practice pending confirmation from larger trials. As noted in the methodological literature, treatment rankings derived from NMA are associated with substantial imprecision (27), and high SUCRA values do not necessarily reflect high-certainty evidence (28).

From a clinical perspective, ROT and ROT-CPT showed point estimates exceeding the commonly used MCID threshold for the FMA-UE (5.2–7.2 points), suggesting potentially meaningful improvements. However, the associated credible intervals crossed the MCID boundary, so clinical significance cannot be confirmed with confidence. Notably, all intervention nodes had mean baseline FMA-UE values between 24.8 and 34.8 (Supplementary Table S3). This range overlaps with the moderate-to-severe impairment population studied by Aaby et al. (29), in whom the distribution-based MCID for the FMA-UE was estimated at only 2.1 points. Because both the minimum detectable change and the MCID vary with baseline motor impairment severity (29), the 5.2–7.2 benchmark may be overly conservative in more severely impaired patients. This observation does not change the threshold used in our primary analysis, but it suggests that a single MCID value may oversimplify clinical interpretation. Accordingly, although VR and BCI interventions did not exceed the commonly used MCID thresholds, their effects may nonetheless carry clinical relevance in more severely impaired patients.

From a rehabilitation practice perspective, robotic interventions may be particularly suitable for delivering intensive and repetitive motor training, which is considered important for post-stroke motor recovery (30). VR-based interventions may enhance patient engagement and motivation by providing interactive and multisensory training environments (31). Although BCI-based rehabilitation has shown promising theoretical potential, its wider clinical implementation remains limited by the very small number of eligible studies (only one RCT), as well as technological complexity, heterogeneous training protocols, and the current limitations of the evidence base (32).

Subgroup analyses suggested that patient characteristics may influence treatment effects; however, these analyses were based on median splits rather than predefined clinical thresholds and should therefore be considered exploratory and hypothesis-generating.

The overall certainty of evidence ranged from low to moderate, primarily due to indirectness and imprecision, as most comparisons relied on indirect evidence within a star-shaped network and involved relatively small sample sizes. Therefore, treatment rankings should be interpreted with caution.

Several limitations of this study should be acknowledged. First, the lack of head-to-head comparisons between different technological interventions limits the strength of indirect comparisons. Additionally, the star-shaped network structure precluded formal assessment of inconsistency, which may affect the reliability of indirect comparisons. Second, many intervention nodes were supported by a small number of studies, with some based on single trials, reducing the reliability of ranking estimates. Third, potential publication bias and small-study effects cannot be excluded due to the limited number of studies per comparison. Finally, heterogeneity in intervention protocols, treatment intensity, and patient characteristics may have influenced the results.

Additionally, although sensitivity analyses excluding high-risk studies yielded consistent directional findings, the limited number of studies per node (most nodes had only 1–2 studies) precludes strong claims of robustness. The term ‘robust’ should be interpreted with caution given the limited evidence base.

In summary, robotic interventions are supported by relatively more consistent evidence for improving upper limb motor function after stroke. In contrast, VR and BCI interventions show potential benefits but remain supported by less robust and more heterogeneous evidence. High-quality, large-scale, head-to-head randomized controlled trials are required to directly compare these technologies and to establish their relative effectiveness in clinical practice.

## 5 Conclusion

In this network meta-analysis of upper-limb rehabilitation after stroke, robotics-based interventions demonstrated the most reliable evidence for effectiveness. Specifically, robotics alone (ROT, 3 studies, SUCRA = 0.71) and robotics combined with conventional physical therapy (CPT) (ROT-CPT, 2 studies, SUCRA = 0.70) showed consistent clinically meaningful improvements, representing the most appropriate recommendations for clinical practice. While a single robotics variant—robotic repetitive facilitative exercise (ROT-RFE)—achieved a numerically higher SUCRA value (0.91), this finding should not be interpreted as definitive evidence of superiority given its single-study basis. Virtual reality interventions demonstrated modest but measurable benefits across 3 studies (SUCRA = 0.62). In contrast, BCI interventions and other robotics variants (ROT-ADL, ROT-HEP, ROT-ITR) were each supported by only one study, limiting reliable clinical conclusions. Overall, the certainty of evidence ranged from low to moderate. High-quality, adequately-powered randomized controlled trials with direct technology comparisons are urgently needed to confirm these findings and guide clinical implementation.

## Data Availability

The original contributions presented in the study are included in the article/Supplementary material, further inquiries can be directed to the corresponding author.

## References

[1] Chen D Shi J Tao B Zhao X Zhao Z Li S . A novel transfer learning-based hybrid EEG-fNIRS brain-computer Interface for intracerebral hemorrhage rehabilitation. Adv Sci (Weinh). (2025) 12:e05426. doi: 10.1002/advs.202505426, 40831229 PMC12631896

[2] Lang CE Macdonald JR Reisman DS Boyd L Jacobson Kimberley T Schindler-Ivens SM . Observation of amounts of movement practice provided during stroke rehabilitation. Arch Phys Med Rehabil. (2009) 90:1692–8. doi: 10.1016/j.apmr.2009.04.005, 19801058 PMC3008558

[3] Olana DD Abessa TG Lamba D Triccas LT Bonnechere B. Effect of virtual reality-based upper limb training on activity of daily living and quality of life among stroke survivors: a systematic review and meta-analysis. J Neuroeng Rehabil. (2025) 22:92. doi: 10.1186/s12984-025-01603-1, 40269877 PMC12020027

[4] Facciorusso S Spina S Filippetti M Reebye R Francisco GE Santamato A. Botulinum toxin combined with robot-assisted therapy for post-stroke spasticity: a systematic review. Toxins (Basel). (2025) 17. doi: 10.3390/toxins17120569, 41441605 PMC12737565

[5] Luo Y Liu X Yang M. Current status and future prospects of brain-computer interfaces in the field of neurological disease rehabilitation. Front Rehabil Sci. (2026) 7:1666530. doi: 10.3389/fresc.2026.1666530, 41743427 PMC12929526

[6] Dias S Welton NJ Sutton AJ Ades AE. NICE Decision Support Unit Technical Support Documents, NICE DSU Technical Support Document 2: A Generalised Linear Modelling Framework for Pairwise and Network Meta-Analysis of Randomised Controlled Trials. London: National Institute for Health and Care Excellence (NICE) (2014). 27466657

[7] Ramos-Murguialday A Broetz D Rea M Läer L Yilmaz O Brasil FL . Brain-machine interface in chronic stroke rehabilitation: a controlled study. Ann Neurol. (2013) 74:100–8. doi: 10.1002/ana.23879, 23494615 PMC3700597

[8] Ballester BR Nirme J Camacho I Duarte E Rodríguez S Cuxart A . Domiciliary VR-based therapy for functional recovery and cortical reorganization: randomized controlled trial in participants at the chronic stage post stroke. JMIR Serious Games. (2017) 5:e15. doi: 10.2196/games.6773, 28784593 PMC5565792

[9] Kiper P Agostini M Luque-Moreno C Tonin P Turolla A. Reinforced feedback in virtual environment for rehabilitation of upper extremity dysfunction after stroke: preliminary data from a randomized controlled trial. Biomed Res Int. (2014):752128. doi: 10.1155/2014/75212 PMC3972918 24745024

[10] Shin JH Ryu H Jang SH. A task-specific interactive game-based virtual reality rehabilitation system for patients with stroke: a usability test and two clinical experiments. J Neuroeng Rehabil. (2014) 11. doi: 10.1186/1743-0003-11-32, 24597650 PMC3975728

[11] Wang A Tian X Jiang D Yang C Xu Q Zhang Y . Rehabilitation with brain-computer interface and upper limb motor function in ischemic stroke: a randomized controlled trial. Med. (2024) 5:559–569.e4. doi: 10.1016/j.medj.2024.02.014, 38642555

[12] Jo K Yu J Jung J. Effects of virtual reality-based rehabilitation on upper extremity function and visual perception in stroke patients: a randomized control trial. J Phys Ther Sci. (2012) 24:1205–8. doi: 10.1589/jpts.24.1205

[13] Sivan M O'Connor RJ Makower S Levesley M Bhakta B. Systematic review of outcome measures used in the evaluation of robot-assisted upper limb exercise in stroke. J Rehabil Med. (2011) 43:181–9. doi: 10.2340/16501977-0674, 21305232

[14] Page SJ Fulk GD Boyne P. Clinically important differences for the upper-extremity Fugl-Meyer scale in people with minimal to moderate impairment due to chronic stroke. Phys Ther. (2012) 92:791–8. doi: 10.2522/ptj.20110009, 22282773

[15] Arya KN Verma R Garg RK. Estimating the minimal clinically important difference of an upper extremity recovery measure in subacute stroke patients. Top Stroke Rehabil. (2011) 18:599–610. doi: 10.1310/tsr18s01-599 22120029

[16] Hiragami S Inoue Y Harada K. Minimal clinically important difference for the Fugl-Meyer assessment of the upper extremity in convalescent stroke patients with moderate to severe hemiparesis. J Phys Ther Sci. (2019) 31:917–21. doi: 10.1589/jpts.31.917, 31871377 PMC6879402

[17] Spineli LM Kalyvas C Papadimitropoulou K. How robust are findings of pairwise and network meta-analysis in the presence of missing participant outcome data? BMC Med. (2021) 19:323. doi: 10.1186/s12916-021-02195-y, 34930276 PMC8691029

[18] Sterne JA Sutton AJ Ioannidis JP Terrin N Jones DR Lau J . Recommendations for examining and interpreting funnel plot asymmetry in meta-analyses of randomised controlled trials. BMJ. (2011) 343:d4002–2. doi: 10.1136/bmj.d4002 21784880

[19] Mehrholz J Pohl M Platz T Kugler J Elsner B. Electromechanical and robot-assisted arm training for improving activities of daily living, arm function, and arm muscle strength after stroke. Cochrane Database Syst Rev. (2018) 2018:Cd006876. doi: 10.1002/14651858.cd006876.pub5, 30175845 PMC6513114

[20] Cramer SC Sur M Dobkin BH O'Brien C Sanger TD Trojanowski JQ . Harnessing neuroplasticity for clinical applications. Brain. (2011) 134:1591–609. doi: 10.1093/brain/awr039, 21482550 PMC3102236

[21] Krakauer JW. Motor learning: its relevance to stroke recovery and neurorehabilitation. Curr Opin Neurol. (2006) 19:84–90. doi: 10.1097/01.wco.0000200544.29915.cc, 16415682

[22] Laver KE Lange B George S Deutsch JE Saposnik G Chapman M . Virtual reality for stroke rehabilitation. Cochrane Database Syst Rev. (2025) 2025:Cd008349. doi: 10.1002/14651858.cd008349.pub5, 40537150 PMC12178777

[23] Soleimani M Ghazisaeedi M Heydari S. The efficacy of virtual reality for upper limb rehabilitation in stroke patients: a systematic review and meta-analysis. BMC Med Inform Decis Mak. (2024) 24:135. doi: 10.1186/s12911-024-02534-y, 38790042 PMC11127427

[24] Levin MF Knaut LA Magdalon EC Subramanian S. Virtual reality environments to enhance upper limb functional recovery in patients with hemiparesis. Stud Health Technol Inform. (2009) 145:94–108. 19592789

[25] Cervera MA Soekadar SR Ushiba J Millán JDR Liu M Birbaumer N . Brain-computer interfaces for post-stroke motor rehabilitation: a meta-analysis. Ann Clin Transl Neurol. (2018) 5:651–63. doi: 10.1002/acn3.544, 29761128 PMC5945970

[26] Salanti G. Indirect and mixed-treatment comparison, network, or multiple-treatments meta-analysis: many names, many benefits, many concerns for the next generation evidence synthesis tool. Res Synth Methods. (2012) 3:80–97. doi: 10.1002/jrsm.1037, 26062083

[27] Trinquart L Attiche N Bafeta A Porcher R Ravaud P. Uncertainty in treatment rankings: reanalysis of network Meta-analyses of randomized trials. Ann Intern Med. (2016) 164:666–73. doi: 10.7326/M15-2521, 27089537

[28] Wang M Wang Y Lei J. Assessing safety and efficacy in a network meta-analysis. J Allergy Clin Immunol Pract. (2022) 10:3047. doi: 10.1016/j.jaip.2022.08.039, 36357054

[29] Aaby D Sullivan JE Carmona C Drogos J Yao J. Minimum detectable change and minimal clinically important difference for the box and block test and Fugl-Meyer assessment in moderate to severe chronic stroke. Arch Rehabil Res Clin Transl. (2026) 8:100623. doi: 10.1016/j.arrct.2026.100623, 42326589 PMC13282828

[30] Chang WH Kim YH. Robot-assisted therapy in stroke rehabilitation. J Stroke. (2013) 15:174–81. doi: 10.5853/jos.2013.15.3.174, 24396811 PMC3859002

[31] Demeco A Zola L Frizziero A Martini C Palumbo A Foresti R . Immersive virtual reality in post-stroke rehabilitation: a systematic review. Sensors (Basel). (2023) 23. doi: 10.3390/s23031712, 36772757 PMC9919580

[32] Mokienko OA Lyukmanov RK Bobrov PD Suponeva NA Piradov MA. Brain-computer interfaces for upper limb motor recovery after stroke: current status and development prospects (review). Sovrem Tekhnologii Med. (2023) 15:63–73. doi: 10.17691/stm2023.15.6.07, 39944367 PMC11811833

[33] Piron L Turolla A Agostini M Zucconi C Cortese F Zampolini M . Exercises for paretic upper limb after stroke: a combined virtual-reality and telemedicine approach. J Rehabil Med. (2009) 41:1016–102. doi: 10.2340/16501977-0459, 19841835

[34] Reinkensmeyer DJ Wolbrecht ET Chan V Chou C Cramer SC Bobrow JE. Comparison of three-dimensional, assist-as-needed robotic arm/hand movement training provided with Pneu-WREX to conventional tabletop therapy after chronic stroke. Am J Phys Med Rehabil. (2012) 91:S232–41. doi: 10.1097/PHM.0b013e31826bce79, 23080039 PMC3487467

[35] Shin JH Bog Park S Ho Jang S. Effects of game-based virtual reality on health-related quality of life in chronic stroke patients: a randomized, controlled study. Comput Biol Med. (2015) 63:92–8. doi: 10.1016/j.compbiomed.2015.03.011, 26046499

[36] Kim WS Cho S Park SH Lee JY Kwon S Paik NJ. A low cost kinect-based virtual rehabilitation system for inpatient rehabilitation of the upper limb in patients with subacute stroke: a randomized, double-blind, sham-controlled pilot trial. Medicine (Baltimore). (2018) 97:e11173. doi: 10.1097/MD.0000000000011173, 29924029 PMC6034563

[37] Kiper P Szczudlik A Agostini M Opara J Nowobilski R Ventura L . Virtual reality for upper limb rehabilitation in subacute and chronic stroke: a randomized controlled trial. Arch Phys Med Rehabil. (2018) 99:834–842.e4. doi: 10.1016/j.apmr.2018.01.023, 29453980

[38] Lee MJ Lee JH Lee SM. Effects of robot-assisted therapy on upper extremity function and activities of daily living in hemiplegic patients: a single-blinded, randomized, controlled trial. Technol Health Care. (2018) 26:659–66. doi: 10.3233/THC-181336, 30124459

[39] Oh YB Kim GW Han KS Won YH Park SH Seo JH . Efficacy of virtual reality combined with real instrument training for patients with stroke: a randomized controlled trial. Arch Phys Med Rehabil. (2019) 100:1400–8. doi: 10.1016/j.apmr.2019.03.013, 31002812

[40] Park M Ko MH Oh SW Lee JY Ham Y Yi H . Effects of virtual reality-based planar motion exercises on upper extremity function, range of motion, and health-related quality of life: a multicenter, single-blinded, randomized, controlled pilot study. J Neuroeng Rehabil. (2019) 16:122. doi: 10.1186/s12984-019-0595-8, 31651335 PMC6813964

[41] Carpinella I Lencioni T Bowman T Bertoni R Turolla A Ferrarin M . Effects of robot therapy on upper body kinematics and arm function in persons post stroke: a pilot randomized controlled trial. J Neuroeng Rehabil. (2020) 17:10. doi: 10.1186/s12984-020-0646-1, 32000790 PMC6990497

[42] Budhota A Chua KSG Hussain A Kager S Cherpin A Contu S . Robotic assisted upper limb training post stroke: a randomized control trial using combinatory approach toward reducing workforce demands. Front Neurol. (2021) 12. doi: 10.3389/fneur.2021.622014, 34149587 PMC8206540

[43] Mekbib DB Debeli DK Zhang L Fang S Shao Y Yang W . A novel fully immersive virtual reality environment for upper extremity rehabilitation in patients with stroke. Ann N Y Acad Sci. (2021) 1493:75–89. doi: 10.1111/nyas.14554, 33442915

[44] Şenocak E Korkut E Aktürk A Ozer AY. Is the robotic rehabilitation that is added to intensive body rehabilitation effective for maximization of upper extremity motor recovery following a stroke? A randomized controlled study. Neurol Sci. (2023) 44:2835–43. doi: 10.1007/s10072-023-06739-3, 36897464

[45] Chang JY Chun MH Lee A Lee A Lee CM. Effects of training with a rehabilitation device (Rebless®) on upper limb function in patients with chronic stroke: a randomized controlled trial. Medicine (Baltimore). (2024) 103:e38753. doi: 10.1097/MD.0000000000038753, 38941364 PMC11466080

[46] Huang Q Jiang X Jin Y Wu B Vigotsky AD Fan L . Immersive virtual reality-based rehabilitation for subacute stroke: a randomized controlled trial. J Neurol. (2024) 271:1256–66. doi: 10.1007/s00415-023-12060-y, 37947856 PMC10896795

[47] Zhang JZ Chen J Liu XL Yan LB Xu SM. Effectiveness of intelligent robotic-assisted training system combined with repetitive facilitative exercise on upper limb motor function after stroke: a randomized controlled trial. BMC Sports Sci Med Rehab. (2025) 17:178. doi: 10.1186/s13102-025-01234-y, 40611355 PMC12224511

[48] Bhattacharjee S Barman A Patel S Sahoo J. The combined effect of robot-assisted therapy and activities of daily living training on upper limb recovery in persons with subacute stroke: a randomized controlled trial. Arch Phys Med Rehabil. (2024) 105:1041–9. doi: 10.1016/j.apmr.2024.01.027, 38367830

[49] Ögün MN Kurul R Yaşar MF Turkoglu SA Avci Ş Yildiz N. Effect of leap motion-based 3D immersive virtual reality usage on upper extremity function in ischemic stroke patients. Arq Neuropsiquiatr. (2019) 77:681–8. doi: 10.1590/0004-282x20190129, 31664343

[50] Lee M Son J Kim J Pyun SB Eun SD Yoon B. Comparison of individualized virtual reality-and group-based rehabilitation in older adults with chronic stroke in community settings: a pilot randomized controlled trial. Eur J Integr Med. (2016) 8:738–46. doi: 10.1016/j.eujim.2016.08.166

[51] Wolf SL Sahu K Bay RC Buchanan S Reiss A Linder S . The HAAPI (home arm assistance progression initiative) trial: a novel robotics delivery approach in stroke rehabilitation. Neurorehabil Neural Repair. (2015) 29:958–68. doi: 10.1177/1545968315575612, 25782693 PMC4573760

