# Supplementary material for: Comparative efficacy of virtual reality, robotics, and brain–computer interface interventions for upper limb rehabilitation after stroke: a systematic review and network meta-analysis
Source: Front Neurol. 2026 Sep 9;17:1882841. doi: 10.3389/fneur.2026.1882841 (PMC13597351; doi:10.3389/fneur.2026.1882841)
Supplement: Supplementary file 1 [file Data_Sheet_1.ZIP › Supplementary_File_S1/Supplementary_Search_Strategies.docx.docx]

Supplementary Search Strategies

Manuscript ID: 1882841

Date of search: 31 October 2025

Search conducted by: Ruichun Pang

1. PubMed

Database: PubMed

Date searched: 31 October 2025

Final Search: #13 OR #14 OR #15

Search	Query	Results

#1	"Stroke"[Mesh]	195,200

#2	"Upper Extremity"[Mesh]	197,479

#3	"Virtual Reality"[Mesh]	9,441

#4	"Robotics"[Mesh]	51,006

#5	"Brain-Computer Interfaces"[Mesh]	5,970

#6	"Randomized Controlled Trials as Topic"[Mesh]	192,080

#7	Strokes OR Cerebrovascular Accident OR Cerebrovascular Accidents OR Cerebral Stroke OR Cerebral Strokes OR Cerebrovascular Apoplexy OR Brain Vascular Accident OR Brain Vascular Accidents OR Cerebrovascular Stroke OR Cerebrovascular Strokes OR Apoplexy OR CVA (Cerebrovascular Accident) OR CVAs (Cerebrovascular Accident) OR Acute Stroke OR Acute Strokes OR Acute Cerebrovascular Accident OR Acute Cerebrovascular Accidents OR acute cerebrovascular lesion OR acute focal cerebral vasculopathy OR apoplectic stroke OR apoplexia OR brain accident OR brain attack OR brain blood flow disturbance OR brain insult OR brain insultus OR cerebral apoplexia OR cerebral insult OR cerebral vascular accident OR cerebral vascular insufficiency OR cerebro vascular accident OR cerebrovascular arrest OR cerebrovascular failure OR cerebrovascular injury OR cerebrovascular insufficiency OR cerebrovascular insult OR cerebrum vascular accident OR cryptogenic stroke OR CVA OR insultus cerebralis OR ischaemic seizure OR ischemic seizure OR stroke OR thrombotic stroke	796,532

#8	Upper Extremities OR Membrum superius OR Upper Limb OR Upper Limbs OR upper extremity	260,181

#9	Educational Virtual Realities OR Educational Virtual Reality OR Instructional Virtual Realities OR Instructional Virtual Reality OR virtual reality	29,564

#10	Remote Operations (Robotics) OR Remote Operation (Robotics) OR Telerobotics OR Soft Robotics OR Soft Robotic OR Socially Assistive Robots OR Socially Assistive Robot OR Social Robots OR Social Robot OR Humanoid Robots OR Humanoid Robot OR Companion Robots OR Companion Robot OR Robotics	122,613

#11	Brain Computer Interfaces OR Neural Interface Technology OR Neural Interface Technologies OR Brain-Computer Interface OR Brain Computer Interface OR Brain-Machine Interfaces OR Brain-Machine Interface OR Brain Machine Interface OR Brain Machine Interfaces OR BCI system (brain-computer interface) OR BCI-controlled neuroprosthetic OR brain computer interface system OR brain computing interface OR brain-computer interfaces OR brain-computer interfacing system OR cerebellum-machine interface OR cerebral-computer interfaces OR direct neural interface OR mind-machine interface	20,131

#12	pragmatic clinical trials as topic OR randomized controlled trials OR randomized controlled trials as topic OR randomized controlled trial (topic) OR Clinical Trials, Randomized OR Trials, Randomized Clinical OR Controlled Clinical Trials, Randomized	929,763

#13	(#1 OR #7) AND (#2 OR #8) AND (#6 OR #12) AND (#3 OR #9)	201

#14	(#1 OR #7) AND (#2 OR #8) AND (#6 OR #12) AND (#4 OR #10)	357

#15	(#1 OR #7) AND (#2 OR #8) AND (#6 OR #12) AND (#5 OR #11)	69

Final combined result: #13 OR #14 OR #15 = 627

2. Web of Science Core Collection

Database: Web of Science Core Collection (SCI: 2009–2025, SSCI: 2009–2025, IC: 1993–2025, CCR: 1985–2025)

Date searched: 31 October 2025

Search	Query	Results

#1	TS=(Strokes OR Cerebrovascular Accident OR Cerebrovascular Accidents OR Cerebral Stroke OR Cerebral Strokes OR Cerebrovascular Apoplexy OR Brain Vascular Accident OR Brain Vascular Accidents OR Cerebrovascular Stroke OR Cerebrovascular Strokes OR Apoplexy OR CVA (Cerebrovascular Accident) OR CVAs (Cerebrovascular Accident) OR Acute Stroke OR Acute Strokes OR Acute Cerebrovascular Accident OR Acute Cerebrovascular Accidents OR acute cerebrovascular lesion OR acute focal cerebral vasculopathy OR apoplectic stroke OR apoplexia OR brain accident OR brain attack OR brain blood flow disturbance OR brain insult OR brain insultus OR cerebral apoplexia OR cerebral insult OR cerebral vascular accident OR cerebral vascular insufficiency OR cerebro vascular accident OR cerebrovascular arrest OR cerebrovascular failure OR cerebrovascular injury OR cerebrovascular insufficiency OR cerebrovascular insult OR cerebrum vascular accident OR cryptogenic stroke OR CVA OR insultus cerebralis OR ischaemic seizure OR ischemic seizure OR stroke OR thrombotic stroke)	388,132

#2	TS=(Upper Extremities OR Membrum superius OR Upper Limb OR Upper Limbs OR upper extremity)	56,507

#3	TS=(Educational Virtual Realities OR Educational Virtual Reality OR Instructional Virtual Realities OR Instructional Virtual Reality OR virtual reality)	46,876

#4	TS=(Remote Operations (Robotics) OR Remote Operation (Robotics) OR Telerobotics OR Soft Robotics OR Soft Robotic OR Socially Assistive Robots OR Socially Assistive Robot OR Social Robots OR Social Robot OR Humanoid Robots OR Humanoid Robot OR Companion Robots OR Companion Robot OR Robotics)	130,341

#5	TS=(Brain Computer Interfaces OR Neural Interface Technology OR Neural Interface Technologies OR Brain-Computer Interface OR Brain Computer Interface OR Brain-Machine Interfaces OR Brain-Machine Interface OR Brain Machine Interface OR Brain Machine Interfaces OR BCI system (brain-computer interface) OR BCI-controlled neuroprosthetic OR brain computer interface system OR brain computing interface OR brain-computer interfaces OR brain-computer interfacing system OR cerebellum-machine interface OR cerebral-computer interfaces OR direct neural interface OR mind-machine interface)	16,698

#6	TS=(pragmatic clinical trials as topic OR randomized controlled trials OR randomized controlled trials as topic OR randomized controlled trial (topic) OR Clinical Trials, Randomized OR Trials, Randomized Clinical OR Controlled Clinical Trials, Randomized)	575,859

#7	#1 AND #2 AND #6 AND #3	330

#8	#1 AND #2 AND #6 AND #4	263

#9	#1 AND #2 AND #6 AND #5	74

Final combined result: #7 OR #8 OR #9 = 667

3. Cochrane Library

Database: Cochrane Library

Date searched: 31 October 2025

Search	Query	Results

#1	MeSH descriptor: [Stroke] explode all trees	18,648

#2	MeSH descriptor: [Upper Extremity] explode all trees	10,909

#3	MeSH descriptor: [Virtual Reality] explode all trees	1,625

#4	MeSH descriptor: [Robotics] explode all trees	2,212

#5	MeSH descriptor: [Brain-Computer Interfaces] explode all trees	132

#6	MeSH descriptor: [Randomized Controlled Trials as Topic] explode all trees	62,383

#7	Strokes OR Cerebrovascular Accident OR Cerebrovascular Accidents OR Cerebral Stroke OR Cerebral Strokes OR Cerebrovascular Apoplexy OR Brain Vascular Accident OR Brain Vascular Accidents OR Cerebrovascular Stroke OR Cerebrovascular Strokes OR Apoplexy OR CVA (Cerebrovascular Accident) OR CVAs (Cerebrovascular Accident) OR Acute Stroke OR Acute Strokes OR Acute Cerebrovascular Accident OR Acute Cerebrovascular Accidents OR acute cerebrovascular lesion OR acute focal cerebral vasculopathy OR apoplectic stroke OR apoplexia OR brain accident OR brain attack OR brain blood flow disturbance OR brain insult OR brain insultus OR cerebral apoplexia OR cerebral insult OR cerebral vascular accident OR cerebral vascular insufficiency OR cerebro vascular accident OR cerebrovascular arrest OR cerebrovascular failure OR cerebrovascular injury OR cerebrovascular insufficiency OR cerebrovascular insult OR cerebrum vascular accident OR cryptogenic stroke OR CVA OR insultus cerebralis OR ischaemic seizure OR ischemic seizure OR stroke OR thrombotic stroke	85,636

#8	Upper Extremities OR Membrum superius OR Upper Limb OR Upper Limbs OR upper extremity	19,854

#9	Educational Virtual Realities OR Educational Virtual Reality OR Instructional Virtual Realities OR Instructional Virtual Reality OR virtual reality	9,238

#10	Remote Operations (Robotics) OR Remote Operation (Robotics) OR Telerobotics OR Soft Robotics OR Soft Robotic OR Socially Assistive Robots OR Socially Assistive Robot OR Social Robots OR Social Robot OR Humanoid Robots OR Humanoid Robot OR Companion Robots OR Companion Robot OR Robotics	2,590

#11	Brain Computer Interfaces OR Neural Interface Technology OR Neural Interface Technologies OR Brain-Computer Interface OR Brain Computer Interface OR Brain-Machine Interfaces OR Brain-Machine Interface OR Brain Machine Interface OR Brain Machine Interfaces OR BCI system (brain-computer interface) OR BCI-controlled neuroprosthetic OR brain computer interface system OR brain computing interface OR brain-computer interfaces OR brain-computer interfacing system OR cerebellum-machine interface OR cerebral-computer interfaces OR direct neural interface OR mind-machine interface	667

#12	pragmatic clinical trials as topic OR randomized controlled trials OR randomized controlled trials as topic OR randomized controlled trial (topic) OR Clinical Trials, Randomized OR Trials, Randomized Clinical OR Controlled Clinical Trials, Randomized	259,085

#13	(#1 OR #7) AND (#2 OR #8) AND (#6 OR #12) AND (#3 OR #9)	61

#14	(#1 OR #7) AND (#2 OR #8) AND (#6 OR #12) AND (#4 OR #10)	77

#15	(#1 OR #7) AND (#2 OR #8) AND (#6 OR #12) AND (#5 OR #11)	24

Final combined result: #13 OR #14 OR #15 = 162

4. Embase

Database: Embase

Date searched: 31 October 2025

Search	Query	Results

#1	'cerebrovascular accident'/exp	528,018

#2	Strokes OR Cerebrovascular Accident OR Cerebrovascular Accidents OR Cerebral Stroke OR Cerebral Strokes OR Cerebrovascular Apoplexy OR Brain Vascular Accident OR Brain Vascular Accidents OR Cerebrovascular Stroke OR Cerebrovascular Strokes OR Apoplexy OR CVA (Cerebrovascular Accident) OR CVAs (Cerebrovascular Accident) OR Acute Stroke OR Acute Strokes OR Acute Cerebrovascular Accident OR Acute Cerebrovascular Accidents OR acute cerebrovascular lesion OR acute focal cerebral vasculopathy OR apoplectic stroke OR apoplexia OR brain accident OR brain attack OR brain blood flow disturbance OR brain insult OR brain insultus OR cerebral apoplexia OR cerebral insult OR cerebral vascular accident OR cerebral vascular insufficiency OR cerebro vascular accident OR cerebrovascular arrest OR cerebrovascular failure OR cerebrovascular injury OR cerebrovascular insufficiency OR cerebrovascular insult OR cerebrum vascular accident OR cryptogenic stroke OR CVA OR insultus cerebralis OR ischaemic seizure OR ischemic seizure OR stroke OR thrombotic stroke	639,342

#3	'upper limb'/exp	435,574

#4	Upper Extremities OR Membrum superius OR Upper Limb OR Upper Limbs OR upper extremity	109,857

#5	'virtual reality'/exp	38,623

#6	Educational Virtual Realities OR Educational Virtual Reality OR Instructional Virtual Realities OR Instructional Virtual Reality OR virtual reality	33,039

#7	'robotics'/exp	52,410

#8	Remote Operations (Robotics) OR Remote Operation (Robotics) OR Telerobotics OR Soft Robotics OR Soft Robotic OR Socially Assistive Robots OR Socially Assistive Robot OR Social Robots OR Social Robot OR Humanoid Robots OR Humanoid Robot OR Companion Robots OR Companion Robot OR Robotics	23,026

#9	'brain computer interface'/exp	12,243

#10	Brain Computer Interfaces OR Neural Interface Technology OR Neural Interface Technologies OR Brain-Computer Interface OR Brain Computer Interface OR Brain-Machine Interfaces OR Brain-Machine Interface OR Brain Machine Interface OR Brain Machine Interfaces OR BCI system (brain-computer interface) OR BCI-controlled neuroprosthetic OR brain computer interface system OR brain computing interface OR brain-computer interfaces OR brain-computer interfacing system OR cerebellum-machine interface OR cerebral-computer interfaces OR direct neural interface OR mind-machine interface	13,345

#11	'randomized controlled trial (topic)'/exp	303,339

#12	pragmatic clinical trials as topic OR randomized controlled trials OR randomized controlled trials as topic OR randomized controlled trial (topic) OR Clinical Trials, Randomized OR Trials, Randomized Clinical OR Controlled Clinical Trials, Randomized	155,111

#13	(#1 OR #2) AND (#3 OR #4) AND (#11 OR #12) AND (#5 OR #6)	103

#14	(#1 OR #2) AND (#3 OR #4) AND (#11 OR #12) AND (#7 OR #8)	72

#15	(#1 OR #2) AND (#3 OR #4) AND (#11 OR #12) AND (#9 OR #10)	35

Final combined result: #13 OR #14 OR #15 = 210

Summary of Search Results

Database	Initial hits	After deduplication

PubMed	627	—

Web of Science	667	—

Cochrane Library	162	—

Embase	210	—

Total	1,666	913 (after Endnote deduplication)
