# Supplementary material for: Comparative efficacy of virtual reality, robotics, and brain–computer interface interventions for upper limb rehabilitation after stroke: a systematic review and network meta-analysis
Source: Front Neurol. 2026 Sep 9;17:1882841. doi: 10.3389/fneur.2026.1882841 (PMC13597351; doi:10.3389/fneur.2026.1882841)
Supplement: Supplementary file 2 [file Data_Sheet_2.ZIP › Supplementary Table/S1.docx]

|  | BCI-CPT | ROT |  | ROT-ADL | ROT-CPT | ROT-HEP | ROT-ITR | ROT-RFE | VR | VR-CPT |  |
| --- | --- | --- | --- | --- | --- | --- | --- | --- | --- | --- | --- |
| BCI-CPT | 0 | -5.22 (-17.47, 6.50) |  | -2.32 (-14.95, 10.02) | -5.36 (-17.41, 7.04) | 0.25 (-20.71, 21.42) | 1.21 (-13.36, 15.72) | -8.77 (-21.57, 3.06) | -3.88 (-15.16, 7.19) | -0.72 (-11.43, 10.33) |  |
| ROT | 5.22 (-6.50, 17.47) | 0 |  | 2.81 (-6.70, 12.60) | 0.02 (-8.79, 8.90) | 5.47 (-13.25, 25.50) | 6.42 (-6.05, 19.05) | -3.74 (-12.89, 5.63) | 1.33 (-6.39, 8.86) | 4.46 (-2.18, 11.58) |  |
| ROT-ADL | 2.32 (-10.02, 14.95) | -2.81 (-12.60, 6.70) |  | 0 | -2.96 (-12.68, 6.74) | 2.59 (-16.74, 22.52) | 3.48 (-9.40, 16.68) | -6.59 (-17.00, 3.33) | -1.65 (-10.53, 7.34) | 1.48 (-6.36, 9.88) |  |
| ROT-CPT | 5.36 (-7.04, 17.41) | -0.02 (-8.90, 8.79) |  | 2.96 (-6.74, 12.68) | 0 | 5.50 (-13.50, 25.07) | 6.39 (-6.00, 18.99) | -3.62 (-13.08, 5.61) | 1.26 (-6.85, 9.35) | 4.42 (-2.34, 11.80) |  |
| ROT-HEP | -0.25 (-21.42, 20.71) | -5.47 (-25.50, 13.25) |  | -2.59 (-22.52, 16.74) | -5.50 (-25.07, 13.50) | 0 | 0.99 (-20.15, 21.50) | -9.24 (-28.85, 10.23) | -4.21 (-23.52, 14.32) | -1.02 (-19.98, 17.64) |  |
| ROT-ITR | -1.21 (-15.72, 13.36) | -6.42 (-19.05, 6.05) |  | -3.48 (-16.68, 9.40) | -6.39 (-18.99, 6.00) | -0.99 (-21.50, 20.15) | 0 | -10.11 (-22.71, 2.58) | -5.09 (-17.20, 6.78) | -1.97 (-13.23, 9.78) |  |
| ROT-RFE | 8.77 (-3.06, 21.57) | 3.74 (-5.63, 12.89) |  | 6.59 (-3.33, 17.00) | 3.62 (-5.61, 13.08) | 9.24 (-10.23, 28.85) | 10.11 (-2.58, 22.71) | 0 | 4.98 (-3.10, 13.92) | 8.09 (0.80, 16.13) |  |
| VR | 3.88 (-7.19, 15.16) | -1.33 (-8.86, 6.39) |  | 1.65 (-7.34, 10.53) | -1.26 (-9.35, 6.85) | 4.21 (-14.32, 23.52) | 5.09 (-6.78, 17.20) | -4.98 (-13.92, 3.10) | 0 | 3.16 (-2.37, 9.32) |  |
| VR-CPT | 0.72 (-10.33, 11.43) | -4.46 (-11.58, 2.18) |  | -1.48 (-9.88, 6.36) | -4.42 (-11.80, 2.34) | 1.02 (-17.64, 19.98) | 1.97 (-9.78, 13.23) | -8.09 (-16.13, -0.80) | -3.16 (-9.32, 2.37) | 0 |  |
