# Supplementary material for: Comparative efficacy of virtual reality, robotics, and brain–computer interface interventions for upper limb rehabilitation after stroke: a systematic review and network meta-analysis
Source: Front Neurol. 2026 Sep 9;17:1882841. doi: 10.3389/fneur.2026.1882841 (PMC13597351; doi:10.3389/fneur.2026.1882841)
Supplement: Supplementary file 2 [file Data_Sheet_2.ZIP › Supplementary Table/S2.docx]

| Comparison | Number of studies | I² | Risk of bias | Inconsistency | Indirectness | Imprecision | Publication bias | Overall quality  (as stated in main text) |
| --- | --- | --- | --- | --- | --- | --- | --- | --- |
| ROT vs CPT | 3 | 0% | Serious | Not serious | Serious | Not serious | Not detected | Moderate |
| VR vs CPT | 3 | 0% | Serious | Not serious | Serious | Serious | Not detected | Low |
| VR-CPT vs CPT | 10 | 52.3% | Serious | Serious | Serious | Not serious | Potential | Low |
| ROT-RFE vs CPT | 1 | NA | Serious | NA | Serious | Very serious | Not detected | Very low |
| ROT-CPT vs CPT | 2 | NA | Serious | Not serious | Serious | Serious | Not detected | Low |
| Other single‑study comparisons | 1 | NA | Serious | NA | Serious | Very serious | Not detected | Very low |
