# Supplementary material for: Comparative efficacy of virtual reality, robotics, and brain–computer interface interventions for upper limb rehabilitation after stroke: a systematic review and network meta-analysis
Source: Front Neurol. 2026 Sep 9;17:1882841. doi: 10.3389/fneur.2026.1882841 (PMC13597351; doi:10.3389/fneur.2026.1882841)
Supplement: Supplementary file 2 [file Data_Sheet_2.ZIP › Supplementary Table/S3.docx]

| **Node** | **Number of studies** | **Total sample size (EG/CG)** | **Mean age (years)** | **Male (%)** | **Stroke stage** | **Baseline FMA‑UE (mean)** | **Total dose (h)** |
| --- | --- | --- | --- | --- | --- | --- | --- |
| CPT | 23 | 572 / 573 | 58.5 | 62% | Acute / subacute / chronic | 28.5 | — |
| ROT | 3 | 47 / 47 | 59.2 | 56% | Subacute / chronic | 32.1 | 18 |
| ROT‑CPT | 3 | 58 / 60 | 55.0 | 66% | Subacute / chronic | 30.5 | 40 |
| ROT‑RFE | 1 | 25 / 25 | 51.0 | 64% | Subacute | 24.8 | 20 |
| ROT‑ADL | 1 | 22 / 22 | 53.3 | 57% | Subacute | 34.8 | 15 |
| ROT‑HEP | 1 | 47 / 45 | 56.9 | 61% | Chronic | 33.7 | 120 |
| ROT‑ITR | 1 | 19 / 22 | 62.0 | 58% | Subacute | 31.4 | 60 |
| VR | 3 | 68 / 64 | 56.8 | 60% | Subacute / chronic | 33.2 | 15 |
| VR‑CPT | 10 | 168 / 168 | 61.0 | 63% | Subacute / chronic | 28.0 | 30 |
| BCI‑CPT | 2 | 166 / 160 | 55.0 | 70% | Subacute / chronic | 27.0 | 20 |

**Notes:** EG = experimental group, CG = control group. Stroke stage was classified according to the original study reports. Total dose refers to the total therapy time of the intervention group (hours).
