# Supplementary material for: Comparative efficacy of virtual reality, robotics, and brain–computer interface interventions for upper limb rehabilitation after stroke: a systematic review and network meta-analysis
Source: Front Neurol. 2026 Sep 9;17:1882841. doi: 10.3389/fneur.2026.1882841 (PMC13597351; doi:10.3389/fneur.2026.1882841)
Supplement: Supplementary file 2 [file Data_Sheet_2.ZIP › Supplementary Table/S4.docx]

| **Study** | **Node** | **Intervention type** | **Experimental group total time** | **Control group total time** | **Dose‑matched?** |
| --- | --- | --- | --- | --- | --- |
| Zhang 2025 | ROT‑RFE | Add‑on | 60 min × 5/wk × 4wk = 1200 min | 60 min × 5/wk × 4wk = 1200 min | Yes |
| Wang 2024 | BCI‑CPT | Add‑on | 60 min × 5/wk × 4wk = 1200 min | 60 min × 5/wk × 4wk = 1200 min | Yes |
| Kiper 2018 | VR‑CPT | Add‑on | 120 min × 5/wk × 4wk = 2400 min | 120 min × 5/wk × 4wk = 2400 min | Yes |
| Lee 2018 | ROT‑CPT | Add‑on | 60 min × 5/wk × 8wk = 2400 min | 60 min × 5/wk × 8wk = 2400 min | Yes |
| Budhota 2021 | ROT‑CPT | Add‑on | 90 min × 3/wk × 6wk = 1620 min | 90 min × 3/wk × 6wk = 1620 min | Yes |
| Mekbib 2021 | VR‑CPT | Add‑on | 120 min × 4/wk × 2wk = 960 min | 120 min × 4/wk × 2wk = 960 min | Yes |
| Huang 2024 | VR‑CPT | Add‑on | 60 min × 5/wk × 3wk = 900 min | 60 min × 5/wk × 3wk = 900 min | Yes |
| Bhattacharjee 2024 | ROT‑ADL | Add‑on | 60 min × 5/wk × 3wk = 900 min | 60 min × 5/wk × 3wk = 900 min | Yes |
| Ögün 2019 | VR | Add‑on | 60 min × 3/wk × 6wk = 1080 min | 60 min × 3/wk × 6wk = 1080 min | Yes |
| Wolf 2015 | ROT‑HEP | Add‑on | 180 min × 5/wk × 8wk = 7200 min | 180 min × 5/wk × 8wk = 7200 min | Yes |
| Shin 2015 | VR‑CPT | Add‑on | 60 min × 5/wk × 4wk = 1200 min | 60 min × 5/wk × 4wk = 1200 min | Yes |
| Carpinella 2020 | ROT | Stand‑alone | 45 min × 5/wk × 4wk = 900 min | 45 min × 5/wk × 4wk = 900 min | Yes |
| Chang 2024 | ROT | Stand‑alone | 30 min × 10 sessions = 300 min | 30 min × 10 sessions = 300 min | Yes |
| Reinkensmeyer 2012 | ROT | Stand‑alone | 60 min × 3/wk × 8‑9wk ≈ 1440‑1620 min | 60 min × 3/wk × 8‑9wk ≈ 1440‑1620 min | Yes |
| Oh 2019 | VR | Stand‑alone | 30 min × 3/wk × 6wk = 540 min | 30 min × 3/wk × 6wk = 540 min | Yes |
| Piron 2009 | VR | Stand‑alone | 60 min × 5/wk × 4wk = 1200 min | 60 min × 5/wk × 4wk = 1200 min | Yes |
| Kiper 2014 | VR‑CPT | Add‑on | 120 min × 5/wk × 4wk = 2400 min | 120 min × 5/wk × 4wk = 2400 min | Yes |
| Park 2019 | VR‑CPT | Add‑on | 60 min × 5/wk × 4wk = 1200 min | 60 min × 5/wk × 4wk = 1200 min | Yes |
| Kim 2018 | VR‑CPT | Add‑on | 60 min × 5/wk × 2wk = 600 min | 60 min × 5/wk × 2wk = 600 min | Yes |
| Lee 2016 | VR‑CPT | Add‑on | 60 min × 3/wk × 8wk = 1440 min | 60 min × 3/wk × 8wk = 1440 min | Yes |
| Ballester 2017 | VR‑CPT | Add‑on | 20‑60 min × 5/wk × 3wk ≈ 300‑900 min | 20‑60 min × 5/wk × 3wk ≈ 300‑900 min | Yes |
| Senocak 2023 | ROT‑ITR | Add‑on | 120 min × 5/wk × 6wk = 3600 min | 120 min × 5/wk × 6wk = 3600 min | Yes |
| Ramos‑Murguialday 2013 | BCI‑CPT | Add‑on | Daily training × 4wk (~20‑30 min/session) | Daily training × 4wk (~20‑30 min/session) | Yes |
| Shin 2014 | VR‑CPT | Add‑on | 40 min × 5/wk × 2wk = 400 min | 20 min × 5/wk × 2wk = 200 min | No |
| Jo 2012 | VR‑CPT | Add‑on | 60 min × 5/wk × 4wk = 1200 min | 30 min × 3/wk × 4wk = 360 min | No |

**Notes:** To control for the confounding effect of total therapy time in the dose‑matched sensitivity analysis, we operationally defined studies as dose‑matched when the intervention and control groups had comparable total therapy time. Studies with clearly imbalanced total therapy time (e.g., Shin 2014 and Jo 2012) were classified as dose‑inequivalent and excluded from the sensitivity analysis. This classification was based on a pre‑specified analytical rule for this sensitivity analysis and should not be interpreted as a validated clinical threshold. The ‘dose‑matched? Yes’ column indicates studies with comparable total therapy time between groups (≤15% difference). For the dose‑matched network meta‑analysis, a subset of these studies was used based on data availability for network estimation (see Supplementary Table S7).
