# Supplementary material for: Comparative efficacy of virtual reality, robotics, and brain–computer interface interventions for upper limb rehabilitation after stroke: a systematic review and network meta-analysis
Source: Front Neurol. 2026 Sep 9;17:1882841. doi: 10.3389/fneur.2026.1882841 (PMC13597351; doi:10.3389/fneur.2026.1882841)
Supplement: Supplementary file 2 [file Data_Sheet_2.ZIP › Supplementary Table/S5.docx]

| **Study** | **D1 (Randomization)** | **D2 (Deviations)** | **D3 (Missing data)** | **D4 (Outcome measurement)** | **D5 (Selective reporting)** | **Overall** |
| --- | --- | --- | --- | --- | --- | --- |
| Piron 2009 | Low | Low | Low | Low | Low | **Low** |
| Reinkensmeyer 2012 | Some concerns | Low | Low | Low | Low | **Some concerns** |
| Ramos‑Murguialday 2013 | Some concerns | Low | Low | High | Low | **High** |
| Kiper 2014 | Low | Low | Low | Low | High | **High** |
| Shin 2014 | Low | High | Low | Low | Low | **High** |
| Shin 2015 | Low | Low | Low | Low | Low | **Low** |
| Kim 2018 | Low | Low | Low | Low | Low | **Low** |
| Kiper 2018 | Low | Low | Low | Low | Low | **Low** |
| Lee 2018 | Low | Low | Low | Low | Low | **Low** |
| Oh 2019 | Some concerns | Low | Low | Low | Low | **Some concerns** |
| Park 2019 | Low | Low | Low | Low | Low | **Low** |
| Carpinella 2020 | Some concerns | Low | Low | Low | Low | **Some concerns** |
| Budhota 2021 | Low | Low | Low | Low | Low | **Low** |
| Mekbib 2021 | Low | Low | Low | Low | Low | **Low** |
| Senocak 2023 | Some concerns | Low | Low | Low | Low | **Some concerns** |
| Chang 2024 | Low | Low | Low | Low | Low | **Low** |
| Huang 2024 | Low | Low | Low | Low | Low | **Low** |
| Zhang 2025 | Low | Low | Low | Low | Low | **Low** |
| Wang 2024 | Low | Low | Low | Low | Low | **Low** |
| Bhattacharjee 2024 | Low | Low | Low | Low | Low | **Low** |
| Ögün 2019 | Some concerns | Low | Low | Low | Low | **Some concerns** |
| Lee 2016 | Some concerns | Low | Low | Low | Low | **Some concerns** |
| Jo 2012 | Some concerns | Low | Low | Low | Low | **Some concerns** |
| Wolf 2015 | Low | Low | Low | Low | Low | **Low** |
| Ballester 2017 | Low | Low | Low | High | Low | **High** |

**Reasons for the four studies judged at high risk of bias:**

| **Study** | **Overall** | **Reason** |
| --- | --- | --- |
| Ramos‑Murguialday 2013 | **High** | Used the cFMA (0‑54) scale, which was converted to FMA‑UE (0‑66); this conversion may have introduced measurement bias (D4). |
| Ballester 2017 | **High** | Outcome assessors were not blinded (D4 high risk). |
| Kiper 2014 | **High** | Selective reporting of outcomes (D5 high risk). |
| Shin 2014 | **High** | Mismatched total therapy time between groups (experimental 40 min/session, control 20 min/session), leading to performance bias (D2 high risk). |
