# Supplementary material for: Comparative efficacy of virtual reality, robotics, and brain–computer interface interventions for upper limb rehabilitation after stroke: a systematic review and network meta-analysis
Source: Front Neurol. 2026 Sep 9;17:1882841. doi: 10.3389/fneur.2026.1882841 (PMC13597351; doi:10.3389/fneur.2026.1882841)
Supplement: Supplementary file 2 [file Data_Sheet_2.ZIP › Supplementary Table/S6.docx]

## **Supplementary Table S6 – Sensitivity analysis: SUCRA rankings by correlation coefficient (r)**

| Intervention | r = 0.3 | r = 0.5 (Primary) | r = 0.7 |
| --- | --- | --- | --- |
| ROT‑RFE | 0.901 | 0.91 | 0.902 |
| ROT | 0.680 | 0.71 | 0.687 |
| ROT‑CPT | 0.698 | 0.70 | 0.696 |
| VR | 0.595 | 0.62 | 0.598 |
| ROT‑ADL | 0.469 | 0.49 | 0.467 |
| ROT‑HEP | 0.380 | 0.39 | 0.368 |
| VR‑CPT | 0.309 | 0.36 | 0.315 |
| BCI‑CPT | 0.321 | 0.35 | 0.328 |
| ROT‑ITR | 0.277 | 0.29 | 0.271 |
| CPT | — | — | — |

**Note:** The sensitivity analyses using r = 0.3 and r = 0.7 were performed by re‑imputing change‑score standard deviations from the original study data. The r = 0.5 column is taken from the primary analysis (Table 3) to ensure consistency with the main results. The SUCRA rankings remained consistent across all three scenarios, with ROT‑RFE, ROT, and ROT‑CPT occupying the top three positions in all cases.
