# Supplementary material for: Comparative efficacy of virtual reality, robotics, and brain–computer interface interventions for upper limb rehabilitation after stroke: a systematic review and network meta-analysis
Source: Front Neurol. 2026 Sep 9;17:1882841. doi: 10.3389/fneur.2026.1882841 (PMC13597351; doi:10.3389/fneur.2026.1882841)
Supplement: Supplementary file 2 [file Data_Sheet_2.ZIP › Supplementary Table/S7.docx]

## **Supplementary Table S7 – Dose‑matched sensitivity analysis results**

| Intervention | SUCRA | MD (vs CPT) | 95% CrI |
| --- | --- | --- | --- |
| ROT‑RFE | 0.907 | 10.20 | (3.51, 16.82) |
| ROT‑CPT | 0.699 | 6.53 | (0.25, 12.73) |
| ROT | 0.694 | 6.43 | (0.43, 12.31) |
| VR | 0.608 | 5.14 | (0.18, 9.85) |
| ROT‑ADL | 0.473 | 3.54 | (–3.69, 10.72) |
| ROT‑HEP | 0.381 | 0.89 | (–17.76, 19.21) |
| BCI‑CPT | 0.326 | 1.10 | (–9.12, 11.34) |
| ROT‑ITR | 0.278 | 0.11 | (–10.72, 10.63) |
| VR‑CPT | 0.266 | 1.20 | (–2.55, 4.36) |
| CPT | — | 0 | Reference |

**Note:** Dose‑matching was defined as ≤15% difference in total therapy time between intervention and control groups. This sensitivity analysis excluded Shin et al. (2014) due to imbalanced therapy time and included 22 studies from the FMA‑UE network. The subset included all 10 intervention nodes; SUCRA rankings remained consistent with the primary analysis, with ROT‑RFE, ROT‑CPT, and ROT occupying the top three positions. For detailed study‑to‑node allocation, see Supplementary Table S4.
